# Long-term exposure to house dust mites accelerates lung cancer development in mice

**DOI:** 10.1186/s13046-022-02587-9

**Published:** 2023-01-21

**Authors:** Dongjie Wang, Wen Li, Natalie Albasha, Lindsey Griffin, Han Chang, Lauren Amaya, Sneha Ganguly, Liping Zeng, Bora Keum, José M. González-Navajas, Matt Levin, Zohreh AkhavanAghdam, Helen Snyder, David Schwartz, Ailin Tao, Laela M. Boosherhri, Hal M. Hoffman, Michael Rose, Monica Valeria Estrada, Nissi Varki, Scott Herdman, Maripat Corr, Nicholas J. G. Webster, Eyal Raz, Samuel Bertin

**Affiliations:** 1 Division of Rheumatology, Allergy and Immunology, Department of Medicine, University of California San Diego, 9500 Gilman Drive, La Jolla, CA 92093-0663 USA; 2 Department of Pharmacology, Institute of Materia Medica, Chinese Academy of Medical Sciences and Peking Union Medical College, Beijing, China; 3 The State Key Laboratory of Respiratory Disease, Guangdong Provincial Key Laboratory of Allergy and Clinical Immunology, Center for Immunology, Inflammation and Immune-Mediated Disease, The Second Affiliated Hospital of Guangzhou Medical University, Guangzhou, China; 4 Division of Gastroenterology and Hepatology, Department of Internal Medicine, Korea University College of Medicine, Seoul, Korea; 5 Networked Biomedical Research Center for Hepatic and Digestive Diseases (CIBERehd), Hospital General Universitario de Alicante, Alicante, Spain; 6 Alicante Institute of Health and Biomedical Research (ISABIAL), Alicante, Spain; 7 Cell IDx Inc, San Diego, CA USA; 8 Division of Pediatric Allergy, Immunology, and Rheumatology, Rady Children’s Hospital of San Diego, University of California San Diego, La Jolla, CA USA; 9 Tissue Technology Shared Resource, Moores Cancer Center, University of California San Diego, La Jolla, CA USA; 10 Department of Pathology, University of California San Diego, 9500 Gilman Drive, La Jolla, CA USA; 11 Division of Endocrinology, Department of Medicine, University of California San Diego, 9500 Gilman Drive, La Jolla, CA USA; 12 Medical Research Service, Veteran Affairs San Diego Healthcare System, San Diego, CA USA

**Keywords:** Lung cancer, Kras, Urethane, House dust mites, Chronic inflammation, NLRP3, IL-1β, CCL2, Macrophages, Tumor microenvironment

## Abstract

**Background:**

Individuals with certain chronic inflammatory lung diseases have a higher risk of developing lung cancer (LC). However, the underlying mechanisms remain largely unknown. Here, we hypothesized that chronic exposure to house dust mites (HDM), a common indoor aeroallergen associated with the development of asthma, accelerates LC development through the induction of chronic lung inflammation (CLI).

**Methods:**

The effects of HDM and heat-inactivated HDM (HI-HDM) extracts were evaluated in two preclinical mouse models of LC (a chemically-induced model using the carcinogen urethane and a genetically-driven model with oncogenic *Kras*^*G12D*^ activation in lung epithelial cells) and on murine macrophages *in vitro*. Pharmacological blockade or genetic deletion of the Nod-like receptor family pyrin domain-containing protein 3 (NLRP3) inflammasome, caspase-1, interleukin-1β (IL-1β), and C–C motif chemokine ligand 2 (CCL2) or treatment with an inhaled corticosteroid (ICS) was used to uncover the pro-tumorigenic effect of HDM.

**Results:**

Chronic intranasal (i.n) instillation of HDM accelerated LC development in the two mouse models. Mechanistically, HDM caused a particular subtype of CLI, in which the NLRP3/IL-1β signaling pathway is chronically activated in macrophages, and made the lung microenvironment conducive to tumor development. The tumor-promoting effect of HDM was significantly decreased by heat treatment of the HDM extract and was inhibited by NLRP3, IL-1β, and CCL2 neutralization, or ICS treatment.

**Conclusions:**

Collectively, these data indicate that long-term exposure to HDM can accelerate lung tumorigenesis in susceptible hosts (e.g., mice and potentially humans exposed to lung carcinogens or genetically predisposed to develop LC).

**Supplementary Information:**

The online version contains supplementary material available at 10.1186/s13046-022-02587-9.

## Background

Lung cancer (LC) is the leading cause of cancer death worldwide [1], with non-small cell lung cancer (NSCLC) accounting for approximately 85% of cases [2]. Several lines of evidence support the hypothesis that individuals with certain chronic inflammatory lung diseases have a higher risk of developing LC independently of their smoking status [3, 4]. Indeed, chronic obstructive pulmonary disease (COPD) is a strong risk factor for LC [5, 6] and an increasing number of studies have demonstrated the positive association between other chronic inflammatory lung diseases and LC [7–11]. Chronic lung inflammation (CLI) as occurring in asthma could increase the risk of developing LC [9, 12, 13] but the exact mechanism by which CLI promotes LC remains unclear. CLI is characterized by diverse molecular and cellular changes, and it is not known which of these changes are essential for the subsequent increased risk of LC. In addition, we do not know which types or subtypes of lung inflammation are causing LC. Therefore, identifying the critical steps by which CLI promotes LC development could provide important new insights into the prevention and treatment of LC.

To better understand the relationship between CLI and LC, we evaluated in this preclinical study the effects of chronic exposure to house dust mites (HDM) in two different mouse models of NSCLC. HDM is a common indoor aeroallergen associated with the development of asthma [14] that can induce CLI and lung epithelial damage [14–17]. Thus, we hypothesized that chronic exposure to HDM could accelerate LC development in susceptible hosts through the induction of pro-inflammatory cytokines such as IL-1β [18–20]. At the cellular level, IL-1β is known to stimulate angiogenesis in the tumor microenvironment (TME) [21], and to induce the production of IL-6 and IL-17A, which are well-established mediators of tumor growth. These cytokines are also involved in the recruitment of tumor-associated macrophages (TAMs) and myeloid-derived suppressor cells (MDSCs) that can promote tumor development and progression [22, 23].

In 2017, the CANTOS (Canakinumab ANti-inflammatory Thrombosis Outcomes Study) clinical trial evaluated the effect of a neutralizing anti-IL-1β antibody (Ab) (canakinumab) in patients with atherosclerosis prone to cardiovascular events and made the serendipitous observation that IL-1β blockade significantly reduced LC incidence and mortality [24]. This study provided the first evidence of anti-IL-1β therapy as a potential treatment for LC and has led to the design of several clinical trials that are currently exploring IL-1β as a therapeutic target in NSCLC [25–27]. However, among the follow-up studies, two recent clinical trials studying the effect of canakinumab in combination with immunotherapy (anti-PD-1 Ab) or chemotherapy in metastatic NSCLC could only demonstrate potential clinical benefits in certain subgroups of patients based on the baseline of inflammatory biomarkers (e.g., C-reactive protein) [27, 28]. Thus, these data support further evaluation of anti-IL-1β therapy in LC and indicate that predictive biomarkers are needed to identify the right patient population.

In this study, we identified that chronic exposure to HDM activates the NLRP3 inflammasome in macrophages, increases the production of IL-1β in the lungs, induces a pro-tumor lung microenvironment, and accelerates LC development and progression in two different mouse models. Together, these data suggest that the effect of HDM is not limited to the induction of allergic lung inflammation and that long-term exposure to HDM may also represent an environmental risk factor for LC.

## Materials and methods

### Animals

Wild-type (WT) C57BL/6 J (JAX, Strain# 000664), *Nlrp3* KO (JAX, Strain# 021302), and *Rag1* KO mice (JAX, Strain# 002216), both on the C57BL/6 J background, were originally purchased from the Jackson Laboratory (JAX). Initial breeding pairs of *Il1b* KO mice (JAX, Strain# 034447) backcrossed to C57BL/6 J for over 10 generations were kindly provided by Dr. Wai Wilson Cheung, Dr. Robert Mak, and Dr. Hal Hoffman (UCSD). Initial breeding pairs of *Casp1* KO mice (JAX, Strain# 016621) on the C57BL/6 background were a gift from Dr. Richard Flavell (Yale University). Initial breeding pairs of CCSP^Cre^ (JAX, Strain# 036525) [29] and *LSL-Kras*^*G12D*^ (JAX, Strain# 008179) [30] mice, both on the C57BL/6 background, were a gift from Dr. Seon Hee Chang (The University of Texas MD Anderson Cancer Center). For the generation of *Kras*^*G12D*^ mice, CCSP^Cre±^ mice were intercrossed with *LSL-Kras*^*G12D±*^ as detailed in the *Kras*^*G12D*^-driven LC model section. All the mice were bred in our vivarium under specific pathogen-free (SPF) or enhanced-barrier SPF (*Rag1* KO) conditions for more than 6 months and were genotyped before they were used in any experiments. All the mice were kept on a 12-h light and 12-h dark cycle with a standard chow diet and water.

### Chemical compounds

Urethane (Sigma-Aldrich, Cat# U2500) was resuspended in 0.9% sodium chloride solution (BD, Cat# 306546) and passed through a 0.22 µm sterile filter before being administered to the mice. Budesonide (Tocris, Cat# 2671) and VX-765 (Selleckchem, Cat# S2228) were reconstituted in DMSO, MCC950 (Selleckchem, Cat# S7809) was resuspended in sterile PBS, and all compounds were aliquoted, and stored at -80 °C until used.

### Allergen extracts

HDM extracts were generated by Greer Laboratories as follows: whole bodies of *Dermatophagoides pteronyssinus* (DP) or *Dermatophagoides farinae* (DF) were extracted with 0.01 M ammonium bicarbonate (1:20, w/v) overnight at 2–8 °C. The crude extract, recovered after centrifugation, was dialyzed against pyrogen-free water, sterilized using a 0.22-µm membrane filter, and lyophilized under aseptic conditions. Lyophilized extracts of HDM DP (Cat# XPB82D3A2.5 or XPB82D3A25) or DF (Cat# XPB81D3A2.5) were resuspended based on their total protein content at 2 mg/mL in sterile 0.9% sodium chloride solution (BD, Cat# 306546) for *in vivo* experiments or at 10 mg/mL in sterile PBS (Thermo Fisher Scientific, Cat# 14190144) for *in vitro* experiments, and were aliquoted and stored at -80 °C until used. When indicated, HDM DP was heat-inactivated (HI-HDM) for 1 h at 95ºC as previously described [31]. HDM and HI-HDM extracts were prepared from the same lot of HDM in all studies comparing their effects *in vitro* or *in vivo*. Other allergens used in this study include *German cockroach* (CR, Cat# XPB46D3A4), *ragweed pollen* (RW, Cat# XP56D3A2.5), *Candida albicans* (CA, Cat# XPM15D3A5), and *Alternaria alternata* (AA, Cat# XPM1D3A2.5). All the allergen extracts were purchased from Greer Laboratories, resuspended based on their total protein content at 10 mg/mL in sterile PBS, and were aliquoted and stored at -80 °C until used.

### Intranasal instillations

In the urethane model, mice were sensitized intranasally (i.n) under light anesthesia (isoflurane) on days 0 and 10 with HDM or HI-HDM (50 μg/50 μL/mouse) or with the control vehicle (VEH; 0.9% sodium chloride, BD, Cat# 306546, 50 μL/mouse), challenged i.n 2x/week for 10 weeks and then 1x/week for an additional 16 weeks with HDM or HI-HDM (12.5 μg/50 μL/mouse) or VEH (50 μL/mouse). In the *Kras*^*G12D*^ model, mice were sensitized i.n on day 0 with HDM, HI-HDM, or OVA (50 μg/30 μL/mouse) or VEH (30 μL/mouse), challenged i.n 2x/week for 4 weeks and 1x/week for 4 weeks with HDM, HI-HDM, or OVA (12.5 μg/30 μL/mouse) or VEH (30 μL/mouse), and then either euthanized (14-week-old time point) of left untreated for an additional 4 weeks (18-week-old time point). When indicated, *Kras*^*G12D*^ mice were treated i.n with budesonide (5 μg/mouse in 30 μL VEH with 0.5% DMSO) or DMSO (30 μL VEH with 0.5% DMSO/mouse) 3x/week and 30 min before each HDM or VEH i.n treatment and then 3x/week for an additional 4 weeks.

### Urethane-induced LC model

Age- and sex-matched C57BL/6 WT or *Il1b* KO mice were injected intraperitoneally (i.p) with urethane (Sigma-Aldrich, Cat# U2500; 1 mg/g of BW) 1x/week for 10 consecutive weeks (tumor initiation stage) followed by a 16-week resting period (tumor promotion stage) as previously described [32]. Due to their observed increased susceptibility to urethane, age- and sex-matched C57BL/6 *Rag1* KO mice were treated with a lower dose of urethane (0.6 mg/g of BW) but with the same number of i.p injections and for the same duration. Twenty-six weeks after the first urethane injection and 72 h after the last i.n challenge, the mice were euthanized by CO2 asphyxiation, and the blood, the lungs, and the BALF were collected. One lung lobe of the right lung was removed, snap-frozen in liquid nitrogen, and stored at -80 °C until further processing. The remaining 4 lung lobes were fixed in 10% buffered formalin for 24 h and stored in histological grade 70% ethanol until paraffin embedding.

### *Kras*^*G12D*^-driven LC model

We crossed the *LSL-Kras*^*G12D*^ strain [30], which carries a Lox-Stop-Lox (LSL) sequence followed by the *Kras*^*G12D*^ point mutation allele commonly associated with human cancer, with a transgenic mouse expressing the Cre recombinase under the control of the Clara cell secretory protein (CCSP) promoter [29], thereby allowing the expression of the mutant KRAS oncogenic protein specifically in lung club cells (formerly known as Clara cells). The resulting CCSP^Cre±^*Kras*^*G12D±*^ mice (hereafter referred to as *Kras*^*G12D*^ mice) were treated from 5 to 14 or 18 weeks of age i.n with HDM, HI-HDM, OVA, or VEH. When indicated, mice were injected i.p with either a neutralizing anti-mouse IL-1β Ab (BioXCell, Cat# BE0246), a neutralizing anti-mouse CCL2 Ab (BioXCell, Cat# BE0185), or with the isotype control Ab (BioXCell, Cat# BE0091) diluted in *InVivo*Pure pH 7.0 Dilution Buffer (BioXCell, Cat# IP0070) and administrated at the same dose (50 μg/100 μL/mouse) and periodicity as previously described [33, 34] or with MCC950 at the dose of 20 mg/kg [35], 1 h before each HDM or VEH i.n treatment and then once a week for an additional 4 weeks. Finally, 24 or 72 h after the last i.n challenge (14-week-old time point) or 4 weeks after the last i.n challenge and 72 h after the last i.p injection (18-week-old time point), the mice were euthanized by CO2 asphyxiation and the blood, the BALF, and the lungs were harvested. After ligation, one lung lobe of the right lung was removed, snap-frozen in liquid nitrogen, and stored at -80 °C until further processing. The remaining 4 lung lobes were fixed by intratracheal instillation and immersion in 10% buffered formalin solution for 24 h and were stored in histological grade 70% ethanol until paraffin embedding.

### BALF cellularity analysis

Mouse lungs were inflated with 1 ml of PBS and BALF was recovered and spun down. Total cell counts were performed manually on a hemocytometer and 1 × 10^5^ cells into 100 μL of PBS/2% fetal bovine serum (FBS) were cytocentrifuged (Cytospin 2, Shandon) onto microscope slides for 3 min at 500 rpm. The air-dried cytospin preparations were stained by Wright-Giemsa stain (Thermo Fisher Scientific) for 3 min at RT and rinsed with deionized water. The slides were mounted in Cytoseal 60 (Thermo Fisher Scientific) and were examined using light microscopy. Differential cell counts were performed according to standard protocols [36].

### Histological analysis

After fixation, the lung samples were brought in histological grade 70% ethanol to the UCSD Moores Cancer Center Tissue Technology Shared Resource for paraffin embedding and sectioning. The fixed lungs were cut into 4–6-μm sections, placed on glass slides, and stained with hematoxylin (Thermo Fisher Scientific, Cat# 7221) and eosin (Thermo Fisher Scientific, Cat# 7111) (H&E) on a Gemini AS slide stainer (Thermo Fisher Scientific) using standard staining procedures. H&E slides were scanned on a Hamamatsu Nanozoomer (Hamamatsu Photonics) or an Aperio AT2 slide scanner (Leica Biosystems), digitized, and whole-slide images were used for tumor assessments. Tumor counts were performed using ObjectiveView (Objective Pathology) or QuPath [37] software in a blinded fashion following expert guidance provided by two board-certified pathologists and current recommendations for the classification of proliferative pulmonary lesions in mice [38]. The tumor multiplicity (i.e., the number of lung lesions per mouse) was calculated on one (*Kras*^*G12D*^ model) or two (urethane model) H&E-stained step sections. (100 μm apart) of 4 lung lobes for each mouse. In the urethane model, ADs and ACs showed ovoid shape. Therefore, we used the following formula to calculate their surface area: major radius x minor radius x π. The tumor area was calculated as the sum of AD and AC surface areas for each mouse and was expressed in mm^2^. Because of the diffuse infiltrative nature of the lung lesions in the *Kras*^*G12D*^ model, the tumor area was determined using Image-Pro Premier (Media Cybernetics) or QuPath software and was expressed as a percentage of the lung surface area or in mm^2^. To evaluate tumor progression in *Kras*^*G12D*^ mice, we employed a 3-stage grading system adapted from [39] with epithelial hyperplasia (EH) and atypical adenomatous hyperplasia (AAH) as grade 1, adenomas (ADs) with well-circumscribed borders and uniform nuclei as grade 2, adenocarcinomas (ACs) with enlarged nuclei, prominent nucleoli, scattered mitotic figures and areas of cell crowding as grade 3. The histological architecture of the lesions was classified as either lepidic (alveolar/bronchiolar hyperplasia), papillary, solid, or mixed papillary and solid (ADs or ACs). The criteria for differentiation between ADs and ACs were practical and were made by size, demarcation of the tumor margins, and cytological atypia [38]. The amount of inflammatory cell infiltrates on H&E-stained lung sections was scored in a blinded fashion using an inflammation score from 0 to 4 adapted from [40]. The inflammation scores refer to absent “0”, very little amount “1” (< 10% of the total surface area of the lungs), little amount “2” (10–25% of the total surface area of the lungs), moderate amount “3” (25–50% of the total surface area of the lungs), and severe amount “4” (> 50% of the total surface area of the lungs) of inflammatory cell infiltrates.

### Immunohistochemistry and immunofluorescence staining

Monoplex immunohistochemistry (IHC) was performed using standard staining procedures as previously described [41]. Briefly, 4–6-μm mouse lung FFPE sections were cleared, dehydrated and antigen retrieval was performed in Antigen Unmasking Solution (Vector Labs, Cat# H-3301) at 95ºC for 30 min. Blocking was done using 3% Donkey serum in TBST for 10 min. The slides were then incubated with a rabbit anti-TTF-1 Ab (Abcam, Cat# ab76013, 1:200 dilution) for 1 h at RT, followed by detection with a goat anti-rabbit IgG-HRP (Cell IDx, Cat# 2RH-100) for 30 min at RT and DAB Chromogen (VWR, Cat# 95041-478) for 5 min. Counterstaining was performed with Mayer’s Hematoxylin (Sigma-Aldrich, Cat# 51275) for 5 min and mounting was done with a xylene-based mountant. For multiplex immunofluorescence (mIF), mouse lung FFPE sections were stained with a custom UltraPlex panel from Cell IDx (San Diego, CA). Briefly, heat-induced epitope retrieval was performed manually using a pressure cooker at a high setting (~ 120ºC) for 15 min in sodium citrate buffer, pH 6.0. All primary and secondary conjugates were created at Cell IDx (see Supplemental Table 1). Exposure times were identical for all samples. Mouse spleen and human tonsil FFPE sections were used as positive and negative controls and were provided by Cell IDx. Each primary Ab-Tag conjugate was previously tested by monoplex and multiplex on mouse spleen and/or human tonsil. Finally, the slides were scanned on an Aperio VERSA (mIF) or an Aperio AT2 (monoplex IHC) (Leica Biosystems). Image adjustments and data analysis were performed using QuPath software. F4/80 and Ki-67 positive cells were quantified using QuPath’s positive cell detection tool following published methods [37] and statistical analysis was performed in Prism (GraphPad Software).

### Flow cytometry analysis

Lung single-cell suspensions were prepared as previously described [42, 43]. Briefly, mice lungs were perfused by cardiac perfusion using 1 mM EDTA containing HBSS after the removal of BALF cells by lavage with PBS. Lung tissues were then incubated at 37 °C for 30 min in the digestion solution (0.5 mg/mL collagenase type IA and 20 μg/mL DNase I in HBSS containing 5% FCS, 100 U/ml penicillin, and 100 μg/mL streptomycin) and the cell suspensions were passed through a 100 μm strainer. Lung single-cells or BMDMs, when indicated, were resuspended at the desired concentration (i.e., 1 × 10^6^ into 100 μL of PBS/2% FBS in 96-well round-bottom plates) in presence of anti-mouse CD16/CD32 (Fc Block, BioLegend, Cat# 101320) Ab and were stained for 30 min at 4ºC in the dark using Abs (all 1:100 dilution) against mouse CD11b (BioLegend, Cat# 101216), CD11c (BioLegend, Cat# 117310), F4/80 (BioLegend, Cat# 123110), Gr-1 (BioLegend, Cat# 108412), and PD-1 (Thermo Fisher Scientific, Cat# 12-9985-81). After surface staining, the cells were washed, fixed in IC Fixation Buffer (Thermo Fisher Scientific, Cat# 00–8222-49), permeabilized in Permeabilization Buffer (Thermo Fisher Scientific, Cat# 00–8333-56), and stained with an Ab (1:150 dilution) against pro-IL-1β (Thermo Fisher Scientific, Cat# 11-7114-80) for 30 min at 4ºC in the dark. Cells were washed and analyzed on an Accuri C6 flow cytometer (BD Biosciences). Data were computed using FlowJo software (Tree Star).

### Immunoblotting

To evaluate protein expression by immunoblot, 30 mg of lung tissue or 1 × 10^6^ BMDMs were homogenized or lysed, respectively, in RIPA buffer (Thermo Fisher Scientific, Cat# 89,900) supplemented with protease inhibitors (MedChemExpress, Cat# HY-K0011) for 5 min on ice. The protein concentration was determined with a protein-quantification kit (Bio-Rad). Protein samples (10 μg/lane) were separated through SDS polyacrylamide gel electrophoresis (4–12% gradient, Invitrogen) and then transferred to PVDF membranes (Millipore). Membranes were blocked with 5% BSA/0.3% Tween 20 in PBS for 45 min at RT and incubated overnight at 4ºC with the following primary Abs (all 1:1000 dilution): Armenian hamster anti-mouse/rat/hamster IL-1β (Santa Cruz Biotechnology, Cat# sc-12742) for lung tissues, rabbit anti-mouse IL-1β (Cell Signaling Technology, Cat# 12426) for BMDMs, mouse anti-mouse/rat caspase-1 p20 (Santa Cruz Biotechnology, Cat# sc-398715), rabbit anti-mouse/human NLRP3 (Cell Signaling Technology, Cat# 15101), and mouse anti-mouse β-Actin (Sigma-Aldrich, Cat# A5316). The blots were washed and incubated for 45 min at RT with their corresponding HRP-conjugated secondary Abs (1:2000 to 1:10,000 dilution): mouse anti-Armenian hamster IgG-HRP (Santa Cruz Biotechnology, Cat# sc-2789), goat anti-rabbit IgG-HRP (Cell Signaling Technology, Cat# 7074), and goat anti-mouse IgG-HRP (BioLegend, Cat# 405306) and developed in SuperSignal West Pico PLUS Chemiluminescent Substrate (Thermo Fisher Scientific, Cat# 34577) according to the manufacturer’s instructions. ChemiDoc Imaging System (Bio-Rad) was used for detection and ImageJ software was used for densitometric measurements.

### In vitro macrophage assays

BMDMs were isolated from WT, *Nlrp3* KO, *Casp1* KO, and *Il1b* KO mice using standard protocols as previously described [43]. Briefly, harvested bone marrow cells were cultured for 6 days in RPMI medium supplemented with 10% FBS, L-Glutamine, 1 × penicillin/streptomycin, and recombinant mouse GM-CSF (BioLegend, Cat# 576,302, 10 ng/mL). After 6 days, the adherent cells were collected, re-plated at 1 × 10^6^ cells per mL in 24- or 96-well plates, and were allowed to adhere to the plates for 24 h. BMDM (CD11b^+^F4/80^+^) purity was routinely checked by flow cytometry and was ~ 80%. BMDMs were then incubated with indicated concentrations of allergens or lipopolysaccharide (LPS, Sigma-Aldrich, Cat# L2630, 100 ng/mL) for 24 h. ATP (Sigma-Aldrich, Cat# A7699, 5 mM) was added to each cell culture well for the last hour of incubation. Following these treatments, the supernatants were collected and cytokine levels were assessed by ELISA. LPS + ATP was used as a positive control for NLRP3 activation and IL-1β release. When indicated, BMDMs were pretreated with a caspase-1 inhibitor (VX-765, Selleckchem, Cat# S2228) or with an NLRP3 inhibitor (MCC950, Selleckchem, Cat# S7809) at concentrations of 10 μM and 100 nM, respectively, for 1 h at 37 °C. RAW 264.7 cells (ATCC, Cat# TIB-71) were cultured in DMEM medium supplemented with 10% FBS and 1 × penicillin/streptomycin and were stimulated with HDM as indicated above for BMDMs.

### RT-qPCR analysis

Isolation of total RNA from lung tissues or BMDMs was carried out with the PureLink RNA Mini Kit (Thermo Fisher Scientific, Cat# 12183018A) following the manufacturer’s protocol. One μg of RNA sample was used for reverse transcription (RT) and cDNA synthesis using qScript cDNA SuperMix (Quanta Biosciences, Cat# 95048). Quantitative real-time PCR (qPCR) was performed on a QuantStudio 3 Real-Time PCR System (Thermo Fisher Scientific, Cat# A28137) using PowerUp SYBR Green Master Mix (Thermo Fisher Scientific, Cat# A25742) according to the manufacturer’s instructions. Samples were run in triplicate and normalized to *Krt19* or *Gapdh* gene expression as indicated in the figure legends. qPCR primers for specific target genes were designed based on their reported sequences and synthesized by Integrated DNA Technologies (IDT) Technologies. See Supplemental Table 2 for a list of the oligonucleotide sequences.

### ELISA

The levels of IL-1β, TNF, or CCL2 in BMDM culture supernatants, in the BALF, or lung tissue homogenates were determined using target-specific ELISA kits (see Supplemental Table 1) and following the manufacturers' instructions.

### Graphical illustrations

Graphical illustrations were created using BioRender (https://biorender.com) and Servier Medical Art (https://smart.servier.com).

### Statistics

Sample sizes for *in vivo* studies were determined based on preliminary studies and the variability of the LC models used. All animal studies were adequately powered to achieve statistically significant results with the smallest number of animals. The number of mice per group and the level of replication for each *in vitro* and *in vivo* experiment are mentioned in the figure legends. Both male and female mice were used in this study. The effects reported were observed in a sex-independent manner and were similar in single- and co-housed mice. Age- and sex-matched mice were randomly assigned to treatment groups. Graphical representations were generated using Prism (GraphPad Software) and data are presented as mean ± SEM. The statistical significance between two groups was determined using unpaired Student t-tests with two-tailed *P*-values. The statistical significance between more than two groups was determined using one- or two-way analysis of variance (ANOVA) with post hoc Bonferroni’s tests. *P*-values of less than 0.05 were considered statistically significant. All statistics were computed using Prism (GraphPad Software).

## Results

### Chronic exposure to HDM accelerates LC development and progression in a urethane-induced LC model

**Fig. 1:**
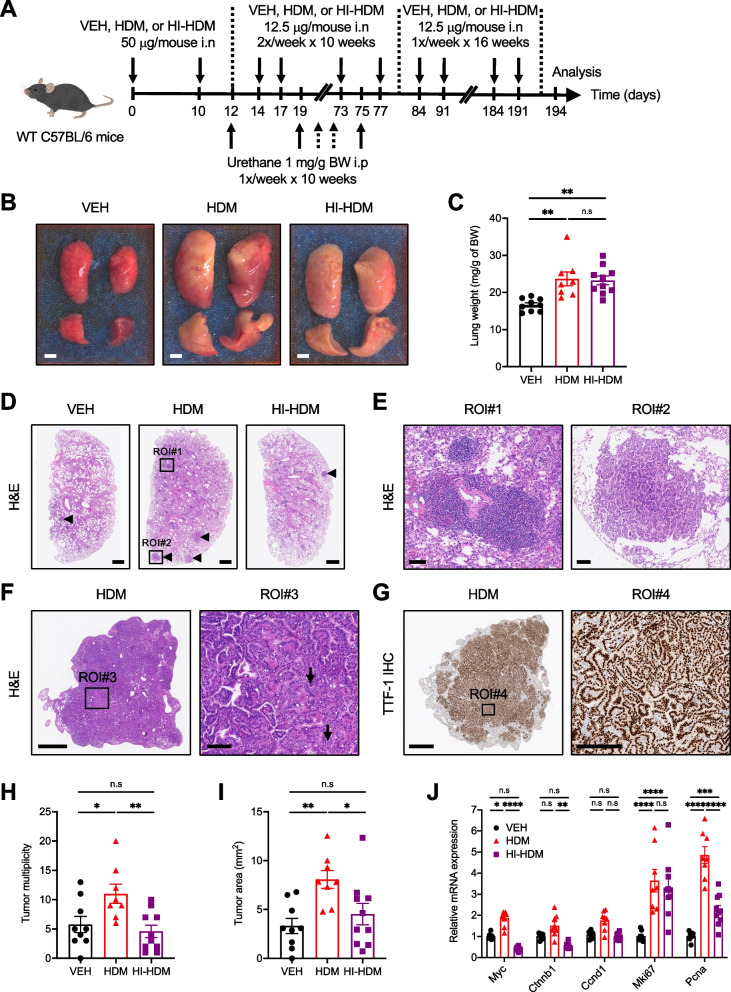
Effect of chronic exposure to HDM in a urethane-induced LC model. **A** WT C57BL/6 mice were treated i.n with HDM (*n* = 8), HI-HDM (*n* = 10), or with the vehicle (VEH, *n* = 9) and i.p with urethane as indicated in this schematic overview of the study design. **B** Representative photos of 4 lung lobes (dorsal view). Scale bars, 0.25 cm. **C** Lung weight normalized to mouse body weight (BW). **D** Representative pictures of H&E-stained lung sections. One lobe per mouse is shown. Arrowheads indicate tumors. Scale bars, 1 mm. **E** Two selected ROIs on the H&E-stained lung section of the mouse treated with urethane and HDM shown in D. ROI#1 shows dense perivascular and peribronchial mononuclear inflammatory infiltrates (left). ROI#2 shows a papillary AD (right). Scale bars, 0.1 mm. **F** Representative photo of an H&E-stained lung section showing an AC of the papillary type found in one lung lobe of a mouse treated with urethane and HDM. The right panel shows the boxed region (ROI#3) at higher magnification with enlarged nuclei, prominent nucleoli, and scattered mitotic figures (indicated by arrows). Scale bars, 1 mm (left panel) and 0.1 mm (ROI#3). **G** IHC staining for TTF-1 of the AC shown in F. The H&E and TTF-1 staining were performed on non-serial sections of the same lung lobe. The right panel shows the boxed region (ROI#4) with strong nuclear staining for TTF-1 in tumor cells at higher magnification. Scale bars, 1 mm (left panel) and 0.2 mm (ROI#4). **H** Tumor multiplicity (i.e., the number of lung lesions per mouse). **I** Tumor area (i.e., the sum of lesion surface areas per mouse). The parameters in H and I were calculated on H&E-stained sections as shown in D. **J** The relative mRNA levels of several genes involved in cell proliferation were analyzed by qPCR in lung homogenates and were normalized to *Krt19* gene expression. The mean expression level of each gene in the VEH group was used as a reference and was assigned the value of 1. Data are representative of one (**G**), two (**J**), or three (**A**–**F**, **H**, and **I**) independent experiments and are presented as mean ± SEM. Statistical significance was assessed by one-way (**C**, **H**, and **I**) or two-way (**J**) ANOVA with post hoc Bonferroni’s test. n.s: non-significant, * P < 0.05, ** *P* < 0.01, *** P < 0.001, **** P < 0.0001

To evaluate the effect of chronic HDM exposure on LC development, we first employed a chemically-induced mouse model of LC using the carcinogen urethane [44]. In both sensitive and resistant strains of mice, urethane induces broncho-alveolar ADs and less frequent ACs with histological features observed in NSCLC [45]. We used a previously established protocol that was shown to overcome the resistance of C57BL/6 mice [32] and examined whether chronic i.n instillation of HDM makes WT C57BL/6 mice more sensitive to urethane-induced lung tumorigenesis. Because HDM is an allergenic mixture that contains multiple proteases that can provoke lung epithelium damage [15], we compared the effects of HDM and heat-inactivated HDM (HI-HDM, 1 h at 95ºC), which denatures its proteins and inactivates its proteolytic activities [31] to those of the control vehicle (VEH) (Fig. 1A). Gross examination of the lungs revealed that mice treated with urethane and HDM or HI-HDM developed lung inflammation as indicated by the enlarged lungs (Fig. 1B) and the increased lung weight (Fig. 1C) as compared to mice treated with urethane and VEH. In addition, mice treated with urethane and HDM demonstrated severe diffuse perivascular/peribronchiolar inflammation with numerous inflammatory cell infiltrates including abundant macrophages surrounding many airways throughout the majority of the lung lobes as shown on H&E-stained lung sections (Fig. 1D) and at higher magnification in the region of interest (ROI) #1 (Fig. 1E). These histopathological features were observed to a lesser degree in mice treated with urethane and HI-HDM and were almost absent in mice treated with urethane and VEH (Fig. 1D and Supplemental Fig. 1A).

We next evaluated lung tumor development in the three experimental groups. Histopathological evaluation of the H&E-stained lung sections revealed that in all the groups, most lesions have the characteristics of alveolar/bronchiolar ADs with predominantly papillary morphology as shown in ROI#2 (Fig. 1E). One hundred percent of the mice treated with urethane and HDM or HI-HDM developed ADs whereas the incidence was 88.9% in mice treated with urethane and VEH. Interestingly, 25% of the mice treated with urethane and HDM also developed ACs characterized by enlarged nuclei, prominent nucleoli, and scattered mitotic figures as shown in ROI#3 (Fig. 1F), which were not observed in mice of the other experimental groups. Immunohistochemistry (IHC) analysis showed that these lesions are positive for thyroid transcription factor-1 (TTF-1), a typical marker of ACs [46] (Fig. 1G). Mice treated with urethane and HDM showed an increased number of lung lesions, which include ADs and ACs, and an increased tumor area as compared to mice treated with urethane and VEH (Fig. 1D, H–I). Interestingly, this was not observed in mice treated with urethane and HI-HDM, which had similar tumor multiplicity and tumor area to that found in VEH-treated mice (Fig. 1D, H–I). In line with these data, we observed an increased expression of several genes involved in cell proliferation such as *Myc*, *Ctnnb1* (encoding the β-catenin protein), *Ccnd1* (encoding the Cyclin D1 protein), *Mki67* (encoding the Ki-67 protein), and *Pcna* in the lungs of mice treated with urethane and HDM compared to mice treated with urethane and VEH or HI-HDM (Fig. 1J). Tumor development was similar in male and female mice (Supplemental Fig. 1B–C). No pleural invasion or distant metastases into other organs were observed in any of the groups (data not shown).

Because inflammation can promote all stages of tumor development, including tumor growth and progression [47], we further evaluated lung inflammation in the different groups. The cellularity in the broncho-alveolar lavage fluid (BALF) was significantly increased in mice treated with urethane and HDM as compared to mice treated with urethane and VEH (Supplemental Fig. 1D–E). Monocytes/macrophages followed by lymphocytes, neutrophils, and eosinophils were the predominant cellular infiltrates in the BALF of HDM-treated mice. HI-HDM-treated mice had a significantly decreased BALF total cell count and showed a reduced trend in BALF differential cell counts (i.e., monocytes/macrophages, lymphocytes, neutrophils, and eosinophils) as compared to HDM-treated mice (Supplemental Fig. 1D–E). However, as previously reported by others [48], heat treatment did not completely abolish the effects of HDM, implying the role of heat-insensitive components in HDM extracts (e.g., heat-insensitive proteases and/or carbohydrates [31, 48]) in the increased BALF cellularity upon HDM exposure. To evaluate the potential role of these different immune cell types in the tumor-promoting effect of HDM, we plotted the BALF total and differential cell counts versus the tumor multiplicity for each mouse of the three experimental groups. Linear regression analyses showed a positive correlation between the number of monocytes/macrophages in the BALF and the tumor multiplicity in mice treated with urethane and HDM but not with urethane and HI-HDM or VEH (Supplemental Fig. 1F). No correlation was found for the other subsets of immune cells in the BALF (data not shown). To evaluate the potential contribution of adaptive immunity to the tumor-promoting effect of HDM, we treated *Rag1* KO mice, which lack mature T and B cells, with urethane and with HDM or VEH as described for WT mice in Fig. 1A, and compared the number of lung lesions in the two different groups. As observed in WT mice (Fig. 1), we found a significantly higher number of lung lesions in *Rag1* KO mice treated with urethane and HDM as compared to *Rag1* KO mice treated with urethane and VEH (Supplemental Fig. 1G–H). Collectively, these results indicate that chronic exposure to HDM accelerates the growth of urethane-induced lung tumors in C57BL/6 mice and that heat-sensitive factors in HDM extracts (e.g., HDM-derived proteases) contribute to this effect. In addition, lung inflammation orchestrated mainly by monocytes/macrophages rather than by adaptive immune cells could mediate the tumor-promoting effect of HDM in this model.

### Chronic exposure to HDM accelerates LC development and progression in a mutant *Kras*-driven LC model

Mutated KRAS is the most common driver mutation in patients with NSCLC and confers a poor prognosis [49]. To confirm the data generated in the urethane-induced LC model and evaluate their potential clinical relevance, we employed CCSP^Cre±^*Kras*^*G12D*±^ mice (hereafter referred to as *Kras*^*G12D*^ mice), which develop spontaneous lung tumors [50] and mirrored KRAS-mutated human ACs [45]. We treated age- and sex-matched *Kras*^*G12D*^ mice i.n with HDM or VEH for 9 weeks, and evaluated lung inflammation as well as the occurrence of lung tumors in the two experimental groups at 14 weeks of age (Supplemental Fig. 2A). At autopsy, the lungs of HDM-treated *Kras*^*G12D*^ mice were larger and weighed more than those of VEH-treated *Kras*^*G12D*^ mice (Supplemental Fig. 2B–C). The cellularity in the BALF of mice treated with HDM was significantly increased compared to VEH-treated mice (Supplemental Fig. 2D). At this time point, both HDM- and VEH-treated *Kras*^*G12D*^ mice developed predominantly alveolar/bronchiolar hyperplasia as shown in ROI#1 (Supplemental Fig. 2E) and fewer ADs and ACs. As observed in the urethane-induced LC model, chronic i.n instillation of HDM significantly increased the tumor multiplicity and the tumor area in *Kras*^*G12D*^ mice as compared to VEH-treated mice (Supplemental Fig. 2E-G). The tumor-promoting effect of HDM was observed in a sex-independent manner and was similar in single- and co-housed *Kras*^*G12D*^ mice (data not shown).

**Fig. 2:**
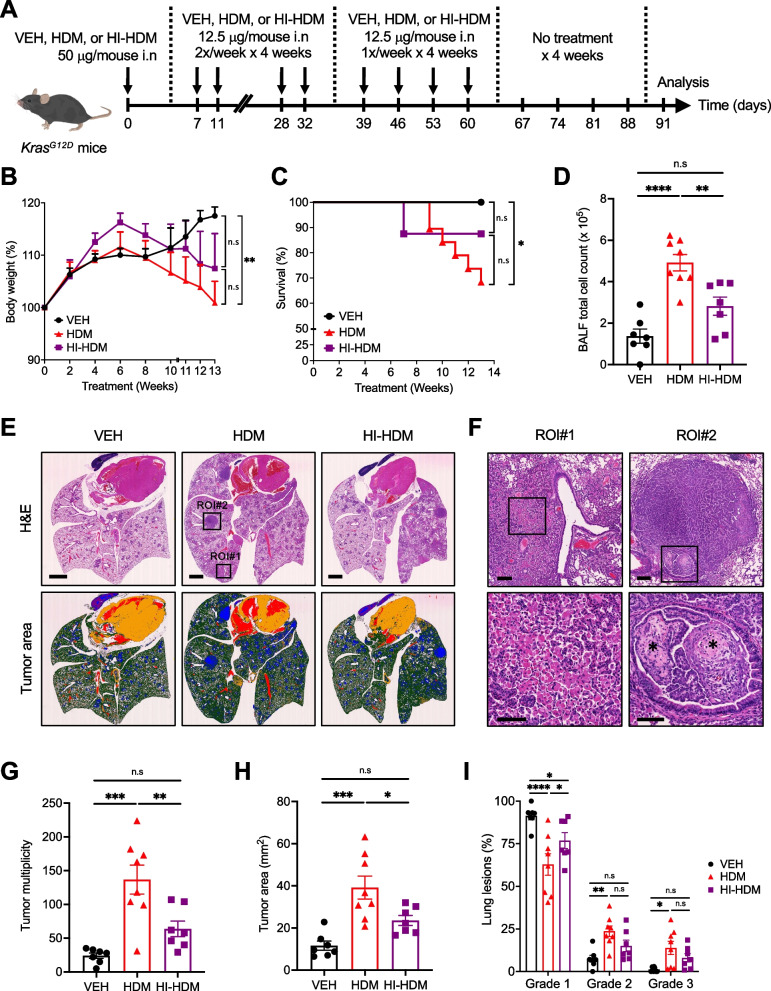
Effect of chronic exposure to HDM in a *Kras*^*G12D*^-driven LC model. **A**. *Kras*^*G12D*^ mice were treated i.n with VEH (*n* = 7), HDM (*n* = 8), or HI-HDM (*n* = 7) as indicated in this schematic overview of the study design. **B** Relative body weight of the mice. **C** Survival curves of mice treated as in A with VEH (*n* = 13), HDM (*n* = 19), or HI-HDM (*n* = 8). Death events occurring within the first 4 weeks after the beginning of the treatments were excluded. Values are expressed as a percentage of survival. **D** BALF total cell counts. **E** Representative pictures of H&E-stained lung sections. The lower panels are the same images as the above panels after tumor area quantification using QuPath software. QuPath-pseudocolored areas are represented as tumors (blue), normal lung cells and infiltrating immune cells (green), heart tissue and eosinophilic cells (orange), blood vessels and areas of hemorrhage (red). Scale bars, 2 mm. **F** Two selected ROIs on the H&E-stained lung section of the HDM-treated mouse shown in E. ROI#1 (left panel) demonstrates dense perivascular and peribronchial immune infiltrates with high numbers of macrophages (inset). ROI#2 (right panel) shows an AC with papillary morphology invading the bronchial lumen (inset) and stromal desmoplasia (indicated by asterisks). Scale bars, 2 mm (whole lungs), 0.2 mm (ROI#1 and 2), and 0.1 mm (ROI#1 and 2 insets). **G** Tumor multiplicity calculated on H&E-stained sections as shown in E upper panels. **H** Tumor area calculated on QuPath-pseudocolored images as shown in E lower panels. **I** Tumors on H&E-stained sections as shown in E upper panels were classified into three grades (Grade 1, AAH and EH; Grade 2, AD; Grade 3, AC) and each grade was expressed as a percentage of the total. Data are presented as mean ± SEM. Statistical significance was assessed by one-way (**D**, **G**, and **H**) or two-way (**B** and **I**) ANOVA with post hoc Bonferroni’s test, or log-rank Mantel-Cox test (**C**). n.s: non-significant, * P < 0.05, ** *P* < 0.01, *** P < 0.001, **** P < 0.0001

To assess the effect of HDM on lung tumor progression and to evaluate the contribution of heat-sensitive factors in HDM extracts in this model, we treated another cohort of *Kras*^*G12D*^ mice i.n with HDM, HI-HDM, or VEH for 9 weeks, let the tumors grow for an additional 4 weeks in absence of i.n treatment and determined the number and subtype of lung tumors in the three experimental groups at 18 weeks of age (Fig. 2A). VEH-treated mice gained weight during the entire protocol whereas mice treated with HDM, and to a lesser degree with HI-HDM, lost weight during the second half of the follow-up period (Fig. 2B). HDM-treated mice had also a decreased survival compared to mice treated with HI-HDM or with VEH in which none of the mice died during the experiment (Fig. 2C). Interestingly, the cellularity in the BALF of mice treated with HDM, and to a lower extent with HI-HDM, remained significantly higher than in VEH-treated mice despite the absence of i.n treatment during the last 4 weeks of the protocol (Fig. 2D). In addition, histological analysis of H&E-stained lung sections revealed the presence of dense perivascular and peribronchial mononuclear inflammatory infiltrates in the lungs of HDM-treated mice (Fig. 2E), with particularly large numbers of macrophages as shown in ROI#1 (Fig. 2F). These inflammatory features were observed to a lesser degree in the lungs of mice treated with HI-HDM and were almost absent in the lungs of VEH-treated mice (Fig. 2E and Supplemental Fig. 2H). At this time point, *Kras*^*G12D*^ mice presented all stages of lung tumor development, from hyperplasia to ACs (Supplemental Fig. 2I). Some tumors in HDM-treated mice demonstrated invasive features, such as invasion of the bronchial lumen and stromal desmoplasia as shown in ROI#2 (Fig. 2F). However, no metastasis was observed in the pleural cavity or distant organs such as the liver, brain, pancreas, and spleen in any of the three experimental groups of mice (data not shown). Mice treated with HDM, but not with HI-HDM, had significantly increased tumor multiplicity and tumor area as compared to mice treated with VEH (Fig. 2G–H). In the three experimental groups, the predominant type of lung lesion was grade 1 (i.e., EH and AAH), followed by the appearance of grade 2 (i.e., ADs) and grade 3 (i.e., ACs) lesions (Fig. 2I, Supplemental Fig. 2I). Interestingly, HDM-treated mice developed significantly less low-grade (i.e., grade 1) but more high-grade (i.e., grades 2 and 3) lesions than VEH-treated mice. This was not observed in mice treated with HI-HDM in which no significant differences were found except for grade 1 lesions as compared to VEH- or HDM-treated mice. However, it is possible that the differences in grade 2 and 3 lesions for HI-HDM-treated mice would have been significant with a higher number of mice per group as increasing the sample size increases statistical power. Collectively, these results suggest that chronic exposure to HDM accelerates the progression of lung lesions from hyperplasia to AD and AC in a *Kras*^*G12D*^-driven mouse model and that heat-sensitive factors in HDM extracts contribute to this effect.

### HDM activates the NLRP3/IL-1β signaling pathway in macrophages

**Fig. 3:**
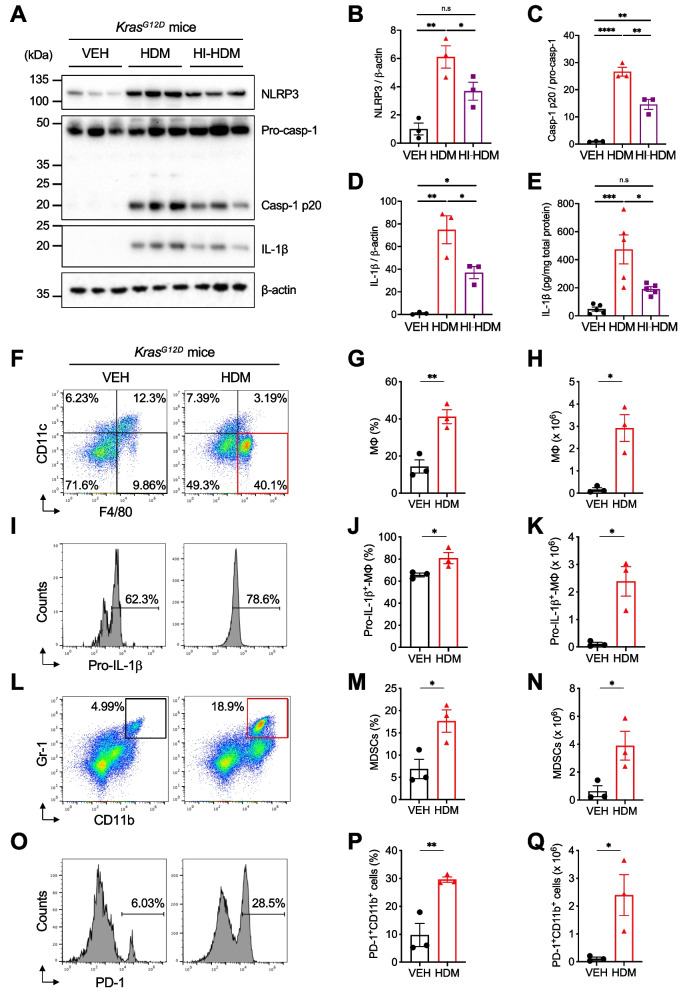
HDM activates the NLRP3/IL-1β signaling pathway in the lungs and generates a pro-tumor lung microenvironment. **A** Lung tissues of *Kras*^*G12D*^ mice treated i.n with VEH, HDM, or HI-HDM, as shown in Fig. 2A, were homogenized for western blot analysis of NLRP3, caspase-1, IL-1β, and β-actin expression (*n* = 3 mice/group). Blots are representative of at least 3 independent experiments. **B** Densitometric measurements of NLRP3, **C**) caspase-1 p20 and **D**) IL-1β, relative to β-actin (B and D) or pro-caspase-1 (C) in the blots shown in A. **E**) ELISA analysis of IL-1β production in lung tissue homogenates as in A (*n* = 5 mice/group). **F-I** The lungs of *Kras*^*G12D*^ mice treated i.n with VEH or HDM as shown in Supplemental Fig. 2A were harvested on day 61 (24 h after the last i.n treatment) and single-cell suspensions were prepared for flow cytometry analyses. **F** Representative dot plots of macrophages (MΦ) identified as CD11b^+^CD11c^–^F4/80^+^ cells (see Supplemental Fig. 3A for gating strategy). **G** Frequencies and **H**) absolute numbers of MΦ as shown in F. **I**) Representative histograms showing intracellular levels of pro-IL-1β in MΦ gated as in F. **J**) Frequencies and **K**) absolute numbers of pro-IL-1β^+^-MΦ as shown in I. **L** Representative dot plots of MDSCs (identified as CD11b^+^Gr-1^hi^ cells). **M** Frequencies and **N**) absolute numbers of MDSCs as shown in L. **O** Representative histograms showing PD-1 expression levels on CD11b^+^ cells (see Supplemental Fig. 3A for gating strategy). **P** Frequencies and **Q**) absolute numbers of PD-1^+^CD11b.^+^ cells as shown in O. Data are representative of one (**F**–**Q**) or two (**A**–**E**) independent experiments and are presented as mean ± SEM. Statistical significance was assessed by one-way ANOVA with post hoc Bonferroni’s test (**A**-**E**) or two-tailed Student’s t-test (**F**-**Q**). n.s: non-significant, * *P* < 0.05, ** *P* < 0.01, *** P < 0.001

Recent studies suggest that activation of the NLRP3 inflammasome and the resulting increase in IL-1β production are associated with tumor progression in various types of cancer, including LC [51]. Therefore, we evaluated the potential activation of the NLRP3/IL-1β signaling pathway in the lungs of *Kras*^*G12D*^ mice treated with HDM, HI-HDM, or VEH. We detected a strong increase in NLRP3 expression (Fig. 3A–B) and caspase-1 cleavage (Fig. 3A, C) as well as increased production of mature IL-1β by immunoblotting (Fig. 3A, D) and by enzyme-linked immunosorbent assay (ELISA) (Fig. 3E) in lung tissue homogenates of *Kras*^*G12D*^ mice treated with HDM, and to a lesser extent with HI-HDM, compared to those of VEH-treated mice. The fact that these parameters remained significantly elevated in the lungs of HDM-treated mice four weeks after the last i.n challenge is consistent with the increased cellularity in the BALF (Fig. 2D) and the inflammatory features observed in the lungs of these mice (Fig. 2E–F ROI#1), and suggest a prolonged activation of the NLRP3/IL-1β signaling pathway by HDM.

Because macrophages are one of the major cell types that express NLRP3 and produce IL-1β [52], we next evaluated by flow cytometry the effect of chronic i.n instillation of HDM on macrophages in the lungs of *Kras*^*G12D*^ mice at the 14-week-old time point, which precedes the development of advanced lung lesions. We observed a significant increase in the frequencies and the absolute numbers of macrophages (identified as CD11b^+^CD11c^–^F4/80^+^ cells as previously described [53, 54]) and macrophages expressing high intracellular levels of pro-IL-1β (Fig. 3I–K) in the lungs of HDM-treated mice as compared to those of VEH-treated mice (Fig. 3F–H, Supplemental Fig. 3A). In addition, the vast majority of the cells in the BALF of HDM-treated mice were positive for the pan-myeloid marker CD11b (Supplemental Fig. 3B–D) and expressed more pro-IL-1β than cells from VEH-treated mice (Supplemental Fig. 3E–G). Interestingly, HDM also induced the accumulation of CD11b^+^ cells expressing high (hi) or intermediate (int) levels of Gr-1 and resembling myeloid-derived suppressor cells (MDSCs, identified as CD11b^+^Gr-1^hi^ cells in lung tissues and CD11b^+^Gr-1^int^F4/80^+^ cells in the BALF) (Fig. 3L–N, Supplemental Fig. 3H–J), and of CD11b^+^ cells expressing programmed cell death protein 1 (PD-1, identified as CD11b^+^PD-1^+^ cells in lung tissues) (Fig. 3O–Q, Supplemental Fig. 3K–M), which are both known to inhibit anti-tumor immunity and to promote tumor development [55, 56]. Collectively, these data suggest that HDM could accelerate lung tumor progression by activating the NLRP3/IL-1β signaling pathway and by creating a pro-tumor lung microenvironment rich in IL-1β^+^-macrophages, MDSCs, and PD-1^+^-myeloid cells.

**Fig. 4:**
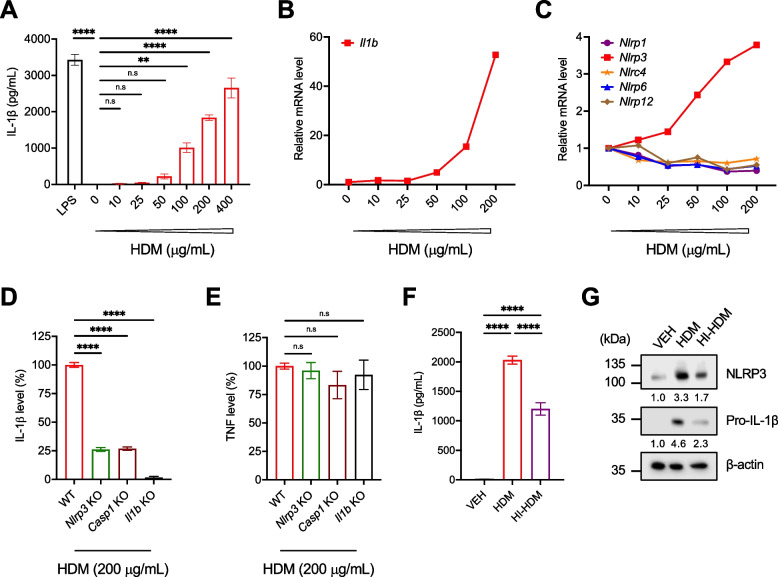
HDM activates the NLRP3 inflammasome and induces IL-1β production by murine macrophages. **A** BMDMs isolated from WT mice were treated for 24 h with LPS (100 ng/mL) or with the indicated concentrations of HDM. ATP (5 mM) was added to each well during the last hour of culture as shown in Supplemental Fig. 4A. The supernatants were collected and IL-1β production was analyzed by ELISA. Results are representative of n = 2 independent experiments performed in triplicate. **B** RAW 264.7 macrophages were stimulated for 6 h with indicated concentrations of HDM and the relative mRNA level of *Il1b* or **C**) different NLRs was measured by qPCR and normalized to *Gapdh* gene expression. The relative mRNA level in the HDM’s 0 μg/mL condition was used as a reference and assigned to 1. Results are representative of *n* = 2 independent experiments performed in simplicate. **D** BMDMs isolated from WT (*n* = 6), *Nlrp3* (*n* = 2), *Casp1* (*n* = 3), or *Il1b* (*n* = 3) KO mice were stimulated with HDM (200 μg/mL) and ATP (5 mM) as shown in Supplemental Fig. 4A, and the levels of IL-1β or **E**) TNF in the supernatants were analyzed by ELISA. The cytokine level in the WT control group was used as a reference and assigned to 100%. Results are the pooled data from *n* = 3 independent experiments performed in triplicate. **F** WT BMDMs were stimulated with VEH (PBS), HDM, or HI-HDM (both at 200 μg/mL) and ATP (5 mM) as described in Supplemental Fig. 4A, and the levels of IL-1β in the supernatants were analyzed by ELISA. Results are representative of *n* = 2 independent experiments performed in triplicate. **G** The cells stimulated in F were recovered and total cell lysates were prepared for western blot analysis of NLRP3 and pro-IL-1β expression. Densitometric measurements of NLRP3 and pro-IL-1β relative to β-actin are indicated below each blot. Results are representative of *n* = 2 independent experiments performed in simplicate. Data are presented as mean ± SEM. Statistical significance was assessed by one-way ANOVA with post hoc Bonferroni’s test. n.s: non-significant, * P < 0.05, ** *P* < 0.01, *** P < 0.001, **** P < 0.0001

To determine whether HDM directly activates the NLRP3/IL-1β signaling pathway in macrophages, we conducted in vitro assays with primary bone marrow-derived macrophages (BMDMs) and with the RAW 264.7 mouse macrophage-like cell line. NLRP3 inflammasome activation requires 2 signals: the priming signal (signal 1), provided by endogenous cytokines and microbial components (e.g., LPS), leads to the activation of nuclear factor-kappa B (NF-κB), and the upregulation of NLRP3 and pro-IL-1β expression; and the activation signal (signal 2), provided by various cellular events and stimuli (e.g., ATP), leads to NLRP3 inflammasome activation and the cleavage of pro-IL-1β into bioactive IL-1β [52]. First, we isolated BMDMs from WT C57BL/6 mice and stimulated them for 24 h with increasing concentrations of HDM or with LPS at the high dose of 100 ng/mL. ATP was added to each well for the last hour of culture and IL-1β secretion in the supernatants was measured by ELISA (Supplemental Fig. 4A). HDM concentration-dependently stimulated IL-1β production by BMDMs up to a level comparable to that obtained with LPS (Fig. 4A). In line with these results and those generated in lung tissues (Fig. 3, A–E), HDM also concentration-dependently stimulated gene expression of *Il1b* and *Nlrp3*, but not *Nlrp1*, *Nlrc4*, *Nlrp6*, or *Nlrp12* in RAW 264.7 macrophages (Fig. 4B–C). Next, we isolated BMDMs from *Nlrp3* and *Casp1* KO mice and found that these cells have significantly reduced IL-1β production after stimulation with HDM (Fig. 4D, Supplemental Fig. 4A). BMDMs isolated from WT and *Il1b* KO mice were used as positive and negative controls, respectively. In contrast, the inflammasome-independent cytokine tumor necrosis factor (TNF) was secreted normally by BMDMs isolated from all three KO mice and stimulated with HDM (Fig. 4E). We obtained similar results by pre-treating WT BMDMs with specific inhibitors of the NLRP3 inflammasome (MCC950) [35] and caspase-1 (VX-765) [57] before HDM stimulation (Supplemental Fig. 4B–C). In agreement with the data generated in lung tissues (Fig. 3A–E), HI-HDM induced significantly lower amounts of IL-1β in BMDM culture supernatants as compared to proteolytically active HDM (Fig. 4F), as well as lower expression levels of NLRP3 and pro-IL-1β in BMDM lysates (Fig. 4G). Together, these data indicate that NLRP3 and caspase-1 are required for HDM-induced IL-1β production by murine macrophages and that heat-sensitive factors in HDM extracts are contributing to this effect.

Finally, we tested whether other common environmental allergens could also induce IL-1β production by macrophages and compared the effect of *Dermatophagoides pteronyssinus* (DP; the HDM species used in this study), *Dermatophagoides farinae* (DF; the second most prevalent HDM species), German cockroach (CR), ragweed pollen (RW), *Candida albicans* (CA) and *Alternaria alternata* (AA) stimulation of BMDMs. Under our experimental conditions (Supplemental Fig. 4A), only DP and to a lesser degree DF and CR triggered IL-1β production by BMDMs (Supplemental Fig. 4D). Interestingly, chronic i.n instillation of DF, but not ovalbumin (OVA), also promoted lung tumor development in *Kras*^*G12D*^ mice (Supplemental Fig. 5). This finding is consistent with the lack of effect of OVA in a urethane-induced LC model [58] and may be imputed to the innocuity of OVA as an antigen.

**Fig. 5:**
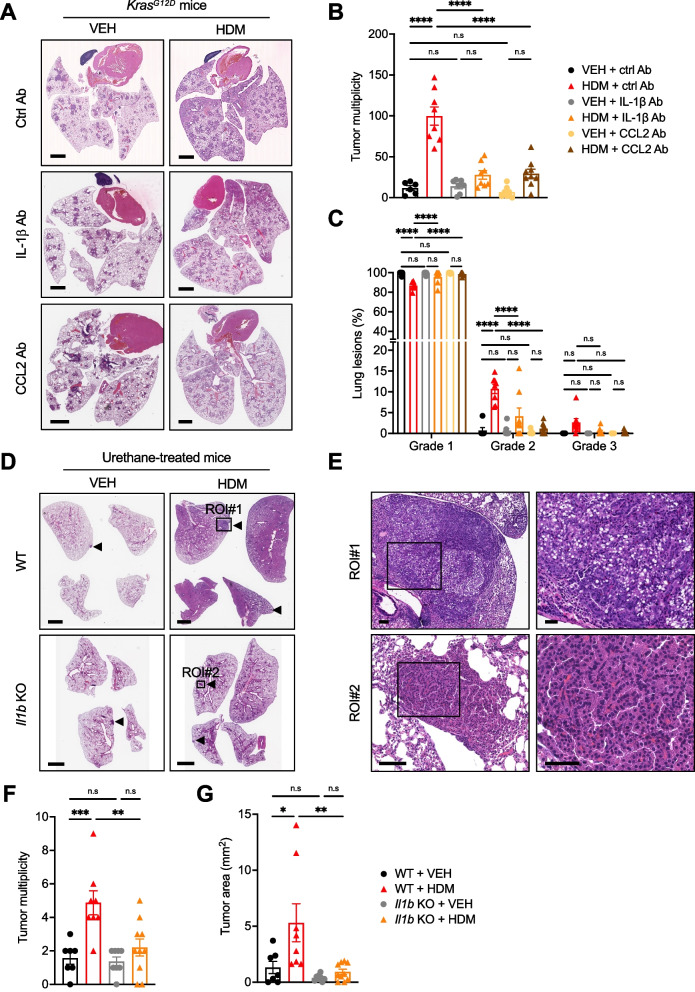
Neutralization of IL-1β or CCL2 inhibits the lung tumor-promoting effect of HDM. **A** Representative pictures of H&E-stained lung sections of *Kras.*^*G12D*^ mice treated i.n with VEH or HDM and i.p with a neutralizing anti-IL-1β, anti-CCL2, or with the isotype control (ctrl) Ab as shown in Supplemental Fig. 5A. VEH + ctrl Ab (*n* = 6), HDM + ctrl Ab (*n* = 8), VEH + IL-1β Ab (*n* = 7), and HDM + IL-1β Ab (*n* = 8), VEH + CCL2 Ab (*n* = 7), and HDM + CCL2 Ab (*n* = 9). Scale bars, 2 mm. **B** Tumor multiplicity calculated on H&E-stained sections as shown in A. **C** Tumors on H&E-stained sections as shown in A were classified into three grades (Grade 1, AAH and EH; Grade 2, AD; Grade 3, AC) and each grade was expressed as a percentage of the total. **D** Representative pictures of H&E-stained lung sections of WT and *Il1b* KO mice treated i.n with VEH or HDM and with urethane as shown in Fig. 1A. WT + VEH (*n* = 7), WT + HDM (*n* = 8), *Il1b* KO + VEH (*n* = 8), and *Il1b* KO + HDM (*n* = 10). Four lobes per mouse are shown. Arrowheads indicate tumors. Scale bars, 1 mm. **E** Two selected ROIs on the H&E-stained lung sections shown in D. ROI#1 shows an AC with enlarged nuclei, prominent nucleoli, and scattered mitotic figures (inset) found in one lung lobe of a WT mouse treated with urethane and HDM. ROI#2 shows an AD of the papillary type with uniform nuclei (inset) found in one lung lobe of an *Il1b* KO mouse treated with urethane and HDM. Scale bars, 0.1 mm (ROI#1 and 2) and 50 μm (ROI#1 and 2 insets). **F** Tumor multiplicity and **G**) Tumor area calculated on H&E-stained sections as shown in D. Data are representative of one experiment conducted in two to five independent cohorts of mice pooled together (**A**–**C** ctrl and CCL2 Abs, and **D**–**G**) or conducted twice independently (**A**–**C** IL-1β Ab) and are presented as mean ± SEM. Statistical significance was assessed by one- (**B**, **F**, and **G**) or two-way (**C**) ANOVA with post hoc Bonferroni’s test. n.s: non-significant, * P < 0.05, ** *P* < 0.01, *** P < 0.001, **** P < 0.0001

### Neutralization of NLRP3, IL-1β, or CCL2 inhibits the tumor-promoting effect of HDM

The role of chronic inflammation in fueling *Kras*-driven LC has been well documented [59]. Because our data indicate that HDM activates the NLRP3 inflammasome in macrophages and induces IL-1β secretion in the lungs, we hypothesized that the NLRP3/IL-1β signaling pathway is the main driver of LC progression in response to chronic HDM exposure in our mouse models. To test this hypothesis, we first evaluated the effect of blocking the NLRP3 inflammasome by treating *Kras*^*G12D*^ mice i.n with HDM or VEH and i.p with MCC950, a potent and selective NLRP3 inhibitor [35] (Supplemental Fig. 6A). At 18 weeks of age, the tumor multiplicity in mice treated with HDM and MCC950 was not significantly different compared to that observed in mice treated with VEH and MCC950 (Supplemental Fig. 6B–C). In addition, both groups of mice had similar percentages of low- and high-grade lesions (Supplemental Fig. 6D). Next, we treated another cohort of *Kras*^*G12D*^ mice treated i.n with HDM or VEH and i.p with an anti-IL-1β Ab or the isotype control Ab and determined the occurrence of lung tumors in the four experimental groups (Fig. 5A, Supplemental Fig. 6A). Although, as previously reported by others, IL-1β neutralization did not affect spontaneous tumor formation in *Kras*^*G12D*^ mice [60], it almost completely abolished the tumor-promoting effect of HDM (Fig. 5A–B). Mice treated with HDM and anti-IL-1β Ab had more grade 1 but fewer grade 2 lesions and also tended to have fewer grade 3 lesions than mice treated with HDM and isotype control Ab (Fig. 5C).

**Fig. 6:**
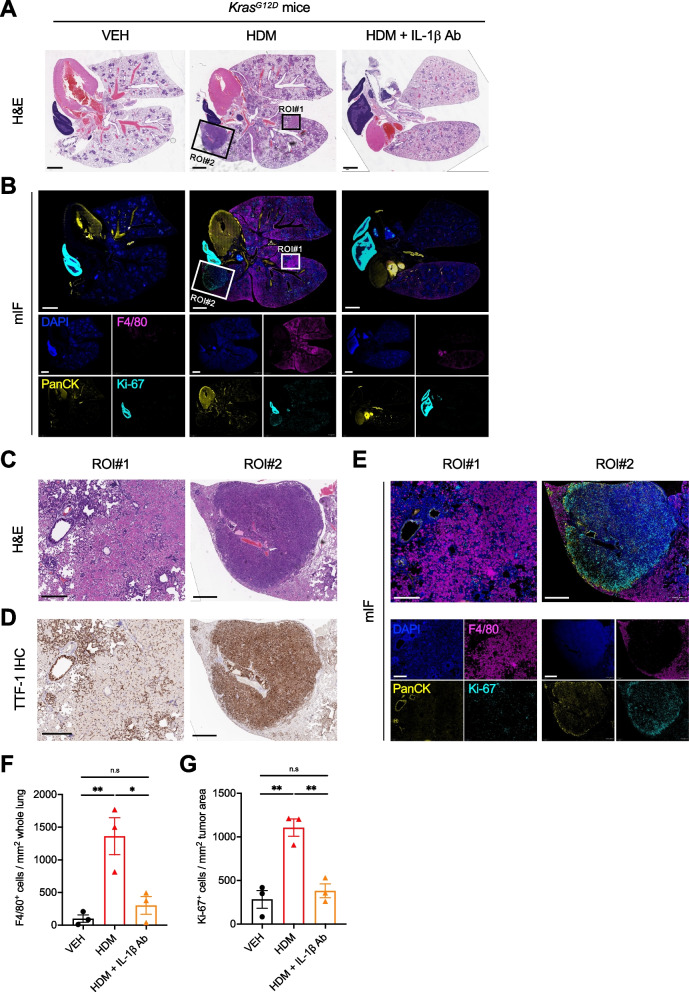
HDM makes the lung TME conducive to tumor growth and IL-1β neutralization abrogates this effect. **A** Representative pictures of H&E-stained lung sections from *Kras*^*G12D*^ mice (*n* = 3 mice/group) treated i.n with VEH or HDM as shown in Fig. 2A, or i.n with HDM and i.p with a neutralizing anti-IL-1β Ab as shown in Supplemental Fig. 6A. The whole lung of one representative mouse from each experimental group is shown. **B** mIF staining of lung sections of the mice shown in A. Overlay (top) and single colors (bottom). DAPI nuclear staining (blue), F4/80 (magenta), Ki-67 (cyan) and PanCK (yellow). **C** Selected ROI#1 (left) and ROI#2 (right) from the H&E-stained lung section of the HDM-treated mouse in A showing inflammatory cell infiltrates and a representative grade 3 lesion, respectively. **D** IHC staining for TTF-1 of the same ROI#1 and ROI#2 as in C. **E** Corresponding mIF images showing high F4/80 immunoreactivity in parenchymal lung tissue (left, ROI#1), and high Ki-67 immunoreactivity in tumor cells as well as peritumoral F4/80^+^ cell infiltration (right, ROI#2). Overlay (top) and single colors (bottom). **F** Quantification of F4/80^+^ cells in whole lungs. **G** Quantification of Ki-67.^+^ cells in tumor areas. Scale bars, 2 mm (whole lungs), 0.4 mm (ROI#1), and 0.8 mm (ROI#2). Data are representative of one (**A**, **C**, and **D**) or two (**B**, **E**–**G**) independent experiments and are presented as mean ± SEM. Statistical significance was assessed by one-way ANOVA with post hoc Bonferroni’s test. n.s: non-significant, * *P* < 0.05, ** *P* < 0.01

To evaluate the effect of IL-1β blockade in the urethane-induced LC model, we treated WT and *Il1b* KO mice i.p with urethane and i.n with HDM or VEH following the protocol shown in Fig. 1A. In line with the effect of IL-1β neutralization in *Kras*^*G12D*^ mice, the tumor multiplicity and the tumor area were strongly reduced in *Il1b* KO mice treated with urethane and HDM as compared to WT mice that received the same treatments and were not significantly different from the tumor multiplicity and tumor area of *Il1b* KO mice treated with urethane and VEH (Fig. 5D, F–G). Interestingly, *Il1b* KO mice treated with urethane and HDM only developed ADs, and none of them developed ACs as observed in WT mice treated with urethane and HDM (Fig. 5E).

Next, we hypothesized that inhibition of CCL2, a key IL-1β-target gene and a main inflammatory chemokine attracting primarily monocytes and macrophages to sites of inflammation [61], would recapitulate the effects of IL-1β blockade in *Kras*^*G12D*^ mice. CCL2 level in the BALF was previously found to correlate with increased macrophage numbers and with lung tumor development in *Kras*^*G12D*^ mice [62]. CCL2 levels were increased in the BALF of *Kras*^*G12D*^ mice treated with HDM, but not with HI-HDM (Supplemental Fig. 7A), and this effect was inhibited by IL-1β blockade (Supplemental Fig. 7B). In line with our hypothesis, CCL2 neutralization almost completely abolished the effect of HDM on tumor development and progression (Fig. 5A–C, Supplemental Fig. 6A). Collectively, these data suggest that the lung tumor-promoting effect of HDM is mostly mediated by the recruitment of macrophages into the lungs and by the activation of the NLRP3/IL-1β signaling pathway in these cells.

**Fig. 7:**
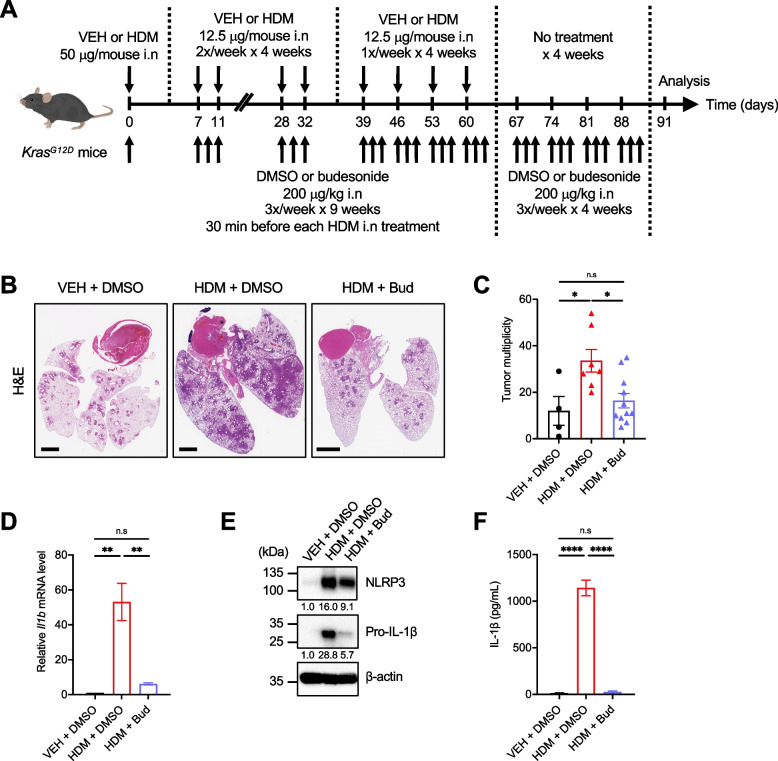
Budesonide inhibits the LC-promoting effect of HDM in *Kras*^*G12D*^ mice and HDM-induced IL-1β production by macrophages. **A***Kras.*^*G12D*^ mice were treated i.n with VEH and dimethyl sulfoxide (DMSO), HDM and DMSO, or HDM and budesonide (Bud) as indicated in this schematic overview of the study design. VEH + DMSO (*n* = 4), HDM + DMSO (*n* = 7), and HDM + Bud (*n* = 11). **B** Representative pictures of H&E-stained lung sections of mice treated as in A. Scale bars, 2 mm. **C** Tumor multiplicity calculated on H&E-stained sections as shown in B. **D** BMDMs were isolated from WT mice as shown in Supplemental Fig. 4A and were treated for 6 h with VEH (PBS) or HDM (200 μg/mL) and DMSO (0.01%**)** or with HDM (200 μg/mL) and budesonide (Bud, 1 nM), and the relative mRNA level of *Il1b* was measured by qPCR and normalized to *Gapdh* gene expression. The relative mRNA level in the VEH condition was used as a reference and assigned to 1. **E** WT BMDMs were treated as in D but for 24 h. ATP (5 mM) was added to each well for the last hour of culture and total cell lysates were prepared for western blot analysis of NLRP3 and pro-IL-1β expression. Densitometric measurements of NLRP3 and pro-IL-1β relative to β-actin are indicated below each blot. **F** The culture supernatants from the cells stimulated in E were recovered and the levels of IL-1β were analyzed by ELISA. Data are representative of two (**A**–**C**) or three (**D**–**F**) independent experiments and are expressed as mean ± SEM. Statistical significance was assessed by one-way ANOVA with post hoc Bonferroni’s test. n.s: non-significant, * P < 0.05, ** *P* < 0.01, **** P < 0.0001

### IL-1β blockade reverses the effects of HDM on the lung tumor microenvironment (TME)

Based on the data presented above, we hypothesized that chronic HDM exposure changes the lung TME and makes it conducive to tumor growth by triggering IL-1β-mediated inflammation. To test this hypothesis, we treaded *Kras*^*G12D*^ mice i.n with VEH or HDM alone or i.n with HDM and i.p with a neutralizing anti-IL-1β Ab, and performed H&E, IHC, and multiplex immunofluorescence (mIF) staining (Fig. 6, Supplemental Fig. 8A–E). In line with the flow cytometry data (Fig. 3F–H), we found a marked increase in F4/80^+^ cells in the lungs of HDM-treated mice as compared to those of VEH-treated mice (Fig. 6B, E–F). As previously described in the *Kras*^*G12D*^-driven LC model [62], we only observed a few intratumoral F4/80^+^ cells, and the vast majority of these cells were found in the parenchymal lung tissue and around the tumors (Fig. 6E). Interestingly, the increased infiltration of F4/80^+^ cells induced by HDM was almost completely abolished by the anti-IL-1β Ab (Fig. 6B, F). Histological analysis revealed that lung tumors of HDM-treated mice express cytokeratins (PanCK), supporting their epithelial origin, and have increased Ki-67 immunoreactivity, indicating their higher proliferating activity, as compared to lung tumors of VEH-treated mice (Fig. 6B, E ROI#2, G). In line with our hypothesis, the anti-IL-1β Ab inhibited the increase in Ki-67^+^ cells in tumors of HDM-treated mice (Fig. 6B, G). Similar to the lung ACs seen in WT mice treated with urethane and HDM (Fig. 1G), strong nuclear staining for TTF-1 was observed in tumor cells of grade 3 lesions from HDM-treated *Kras*^*G12D*^ mice (Fig. 6D, Supplemental Fig. 8F). Collectively, these data indicate that chronic exposure to HDM induces a lung TME rich in macrophages and increases tumor cell proliferation in an IL-1β-dependent manner.

### Budesonide treatment inhibits the pro-tumorigenic effect of HDM

Because IL-1β and CCL2 contribute to the development of CLI, which may mediate the link between chronic exposure to HDM in asthmatic patients and an increased risk of developing LC [8, 10], we tested in a last set of experiments the effect of budesonide, an ICS commonly prescribed to reduce airway inflammation in asthmatic and COPD patients [63]. In addition to its anti-inflammatory effect, budesonide was shown to have anti-proliferative properties [64] and may decrease the risk of developing LC in asthmatic and COPD patients [65, 66]. Consistent with its chemopreventive effect in different mouse models of LC [67–69], budesonide i.n treatment inhibited the tumor-promoting effect of HDM in *Kras*^*G12D*^ mice (Fig. 7A–C). Lastly, we determined the effect of budesonide treatment on NLRP3 activation and IL-1β production using BMDMs. Budesonide strongly inhibited HDM-induced *Il1b* mRNA (Fig. 7D) and pro-IL-1β expression (Fig. 7E) as well as mature IL-1β secretion (Fig. 7F), even though budesonide only slightly inhibited HDM-induced NLRP3 upregulation (Fig. 7E) at the low-dose of 1 nM and at the time point chosen in this assay. Overall, these findings are in agreement with previous studies using budesonide [70, 71] or other ICS [72, 73] and suggest that ICS treatment decreases lung tumor development in mice and might reduce the risk of developing LC in asthmatic and COPD patients, at least in part, by inhibiting the NLRP3/IL-1β signaling pathway in lung macrophages.

## Discussion

Despite recent advances in the prevention and treatment of LC, the exact mechanisms by which CLI promotes LC development remain unclear. To fill this gap of knowledge, we tested the hypothesis that chronic exposure to HDM, a major cause of allergic asthma, can provoke CLI and consequently, accelerate the development of LC in mice. We report here for the first time that the pathological effect of HDM is not limited to the induction of allergic lung inflammation as it occurs in asthma, and that chronic exposure to HDM also accelerates LC development and progression in two different mouse models of LC.

Mechanistically, we identified that the chronic activation of the NLRP3/IL-1β signaling pathway is a possible underlying mechanism (see Supplemental Fig. 9 for the proposed model) as neutralization of NLRP3 or IL-1β, almost completely abolished the LC-promoting effect of HDM. Interestingly, NLRP3 activation, IL-1β production, and the pro-tumorigenic effect of HDM were significantly decreased but not completely abolished by heat treatment of the HDM extract. As published by others using a similar heat-inactivation method and HDM extract from Greer Laboratories it could potentially be due to the remaining protease activity in the HI-HDM extract (28% protease activity remaining after 1 h at 95ºC [31]). It is also possible that both heat-sensitive and -insensitive factors contributed to the observed effects. For example, HDM-derived proteases such as Der p1 (*D. pteronyssinus* allergen 1) and Der f1 (*D. farinae* allergen 1) were shown to directly activate NLRP3 in human bronchial epithelial cells and to induce caspase-1-mediated IL-1β secretion [74, 75]. LPS in HDM extracts could also contribute to NLRP3 activation and IL-1β production [52], and chronic exposure to high doses of LPS was shown to promote lung tumorigenesis in the nicotine-derived nitrosamine ketone (NNK)- and benzo[a]pyrene (B[a]P)-induced LC models [76, 77]. In addition, chitin present in HDM extracts and chitosan, which is derived from deacetylated chitin were shown to activate NLRP3 and induce IL-1β production [48, 78, 79], and may therefore contribute to the effects observed in response to HDM exposure. However, the biochemical complexity of HDM makes challenging investigations on the contribution of a given component to the observed effects, and further studies are required to identify the active component(s) in HDM responsible for its pro-tumorigenic effect. In addition, our results suggest that HDM predominantly activates the NLRP3 inflammasome in macrophages, but we cannot exclude that other cell types such as neutrophils and/or eosinophils also contribute to IL-1β secretion and to the tumor-promoting effect of HDM.

Interestingly, the tumor-promoting effect of HDM was more pronounced in the *Kras*^*G12D*^-driven model (effect size of ~ 4–5/VEH group) than in the urethane-induced model (effect size of ~ 2–3/VEH group). A potential explanation could be that urethane-induced tumors in WT mice carry mostly *Kras*^*Q61L/R*^ mutations [80, 81], which could potentially be less sensitive to the effect of lung inflammation and to a proinflammatory TME than the *Kras*^*G12D*^ mutation [59, 82]. In support of this hypothesis, a recent study shows that IL-1β blockade decreases *Kras*^*G12D*^-induced lung tumorigenesis by shifting the immunosuppressive TME to an antitumor phenotype, possibly via modulating the NF-κB and signal transducer and activator of transcription 3 (STAT3) pathways [83]. Thus, chronic activation of the NLRP3/caspase-1/IL-1β signaling pathway by HDM may mediate a positive feedback loop that amplifies oncogenic *Kras*^*G12D*^ activity. Our results, therefore, suggest that the effect of HDM varies in presence of different driver mutations, and further studies are needed to test the effects of HDM in other oncogene-driven LC models (e.g., epidermal growth factor receptor (EGFR)-mutant LC model as EGFR is one of the most frequently mutated driver gene in LC, particularly in non-smokers [84, 85]).

The relationship between allergic diseases and cancer is a very controversial topic, widely discussed during the last decades, which has led to the emerging interdisciplinary field of AllergoOncology [86]. Indeed, some epidemiological studies have demonstrated an inverse association between allergy and cancer, but others have reached neutral conclusions or have indicated a positive role of allergy in the development of cancer. Epidemiological studies are difficult to perform in this field because of the many confounding factors. For example, asthma was found to be protective against the risk of developing LC in some studies potentially because asthmatics tend to be non-smokers [87]. Asthma’s relationship to LC is still controversial, however, the recent discovery of different asthma endotypes Th2/Th17-low and Th2/Th17-predominant, in which IL-1β plays a critical role [88], may explain this controversy and help the design of future epidemiological studies that will take into account the heterogeneity of asthma. Another potential reason why some epidemiological studies did not identify a positive association between asthma and the risk of LC could be because most asthmatic patients are treated with an ICS, which has anti-inflammatory and anti-proliferative properties, and may decrease the risk of developing LC [65, 66, 89]. Indeed, in a recent study including 75,307 participants, adults with asthma had a 2.8-fold increased risk of developing LC, and treatment with ICS was associated with a 56% risk reduction [11], suggesting that an inflammatory response mediates the link between asthma and LC. In agreement with a previous study [70], we found that budesonide inhibits NLRP3 and decreases IL-1β production by macrophages, which could explain, at least in part, its anti-tumor effect.

## Conclusions

Our study demonstrates a causal link between chronic exposure to HDM and the acceleration of lung tumorigenesis in mice. These data indicate that the effect of long-term exposure to HDM goes beyond the induction of asthma and allergic lung inflammation, and increases the risk of developing LC in susceptible animals. Thus, one can speculate that human subjects chronically exposed to HDM, who do not develop asthma and therefore do not take ICS, are more vulnerable to the LC-promoting effect of HDM. However, further studies are needed to test the effects of naturally occurring HDM exposure, which includes whole mite bodies, feces, and associated bacteria, and determine whether long-term exposure to HDM represents an environmental risk factor for LC in humans. 

## Supplementary Information

**Additional file 1: Supplemental Fig. 1.** Effect of chronic exposure to HDM in a urethane-induced LC model. A-F) WT C57BL/6 mice were treated i.n with HDM (*n* = 8), HI-HDM (*n* = 10), or with the vehicle (VEH, *n* = 9) and i.p with urethane as shown in Fig. 1A. A) Quantification of inflammatory cell infiltrates on H&E-stained lung sections as shown in Fig. 1D. B) The tumor multiplicity and C) the tumor area data presented in Fig. 1H–I were subdivided into male and female mice for the three experimental groups. D) Representative images of Wright-Giemsa staining of BALF cytospins. Magnification, 200x (overview panels), 400x (insets). E) BALF total and differential cell counts of BALF cytospins as shown in D. F) Correlation analysis of the number of monocytes/macrophages in the BALF with tumor multiplicity. The Pearson coefficient (R squared) and the corresponding *P*-value are shown. G) Representative pictures of H&E-stained lung sections of *Rag1* KO mice treated i.n with VEH (*n* = 9) or HDM (*n* = 9), and i.p with urethane (0.6 mg/g of BW) as shown in Fig. 1A for WT mice. One lobe per mouse is shown. Arrowheads indicate tumors. Scale bars, 1 mm. H) Tumor multiplicity calculated on H&E-stained sections as shown in G. Data are presented as mean ± SEM. Statistical significance was assessed by one-way (A) or two-way (B, C, and E) ANOVA with post hoc Bonferroni’s test, linear regression using Pearson correlation (F), or two-tailed Student’s t-test (H). n.s: non-significant, * *P* < 0.05, ** *P* <0.01, *** *P* <0.001, **** *P* < 0.0001. **Supplemental Fig. 2.** Effect of chronic exposure to HDM in a *Kras*^*G12D*^-driven LC model. A) Five-week-old *Kras*^*G12D*^ mice were treated i.n with VEH (n = 11) or HDM (n = 12) as indicated in this schematic overview of the study design. At 14 weeks of age and 72h after the last HDM challenge the mice were sacrificed and the BALFs and the lungs were harvested. B) Representative pictures of 4 lung lobes (dorsal view) of a VEH- and an HDM-treated mouse. Scale bars, 0.25 cm. C) Lung weight normalized to mouse body weight (BW). D) BALF total cell counts. E) Representative pictures of H&E-stained lung sections. The lower panels are the same images as the above panels after tumor area quantification using QuPath software. The area within the blue borders was considered positive for lung tumors. The right panels show the boxed region (ROI#1) at higher magnification with areas of alveolar/bronchiolar hyperplasia and numerous mononuclear inflammatory infiltrates. Scale bars, 2 mm (whole lungs) and 0.5 mm (ROI#1). F) Tumor multiplicity calculated on H&E-stained sections as shown in E upper panels. G) Tumor area calculated on H&E-stained sections as shown in E lower panels. H) Quantification of inflammatory cell infiltrates on H&E-stained lung sections of 18-week-old *Kras*^*G12D*^ mice treated i.n with VEH, HDM, or HI-HDM as shown in Fig. 2E. I) Representative pictures of H&E-stained lung sections showing the different types of lung lesions observed in 18-week-old *Kras*^*G12D*^ mice treated i.n with VEH, HDM, or HI-HDM as shown in Fig. 2E. Top left panels: Grade 1 lesion of AAH with lepidic growth pattern observed in a *Kras*^*G12D*^ mouse treated with VEH. Scale bars, 0.1 mm (overview panel and inset). Middle left panels: Grade 1 lesion of EH of a respiratory bronchiole with hyperproliferative cells in the alveolar compartment observed in a *Kras*^*G12D*^ mouse treated with VEH. Scale bars, 0.1 mm (overview panel and inset). Middle right panels: Grade 2 AD of the papillary type with uniform nuclei observed in a *Kras*^*G12D*^ mouse treated with VEH. Scale bars, 0.2 mm (overview panel) and 0.1 mm (inset). Right panels: Grade 3 AC of the papillary type with enlarged nuclei, prominent nucleoli, and some areas of tumor cell crowding (field of cells indicated by arrows) observed in a *Kras*^*G12D*^ mouse treated with HDM.Scale bars, 0.25 mm (overview panel) and 0.1 mm (inset).Data are representative of one experiment conducted in six independent cohorts of mice pooled together and are presented as mean ± SEM. Statistical significance was assessed by two-tailed Student’s t-test (C, D, F, and G) or one-way ANOVA with post hoc Bonferroni’s test (H). ** *P* <0.01, *** *P* <0.001, **** *P* <0.0001. **Supplemental Fig. 3.** Effect of chronic HDM exposure on the lung microenvironment. *Kras*^*G12D*^ mice were treated i.n with VEH or HDM (*n* = 3 mice/group) as shown in Fig. 2A and were sacrificed at day 61 (24h after the last i.n treatment). The lungs and the BALFs were recovered and single-cell suspensions were prepared for flow cytometry analyses. A) Representative flow cytometry plots of an HDM-treated mouse illustrating the gating strategy for the flow cytometry analyses. First, dead cells and debris were excluded based on forward scatter area (FSC-A) and side scatter area (SSC-A) values, then live single cells were gated based on forward scatter height (FSC-H) and FCS-A values.Macrophages (MΦ) were then identified as CD11b^+^CD11c^–^F4/80^+^ cells. B) Representative dot plots of cells expressing the pan-myeloid marker CD11b in the BALFs of VEH- and HDM-treated mice. C) Frequencies and D) absolute numbers of CD11b^+^ cells as shown in B. E) Representative histograms showing intracellular levels of pro-IL-1β in CD11b^+^ cells gated as in B in the BALFs of VEH- and HDM-treated mice. F) Frequencies and G) absolute numbers of pro-IL-1β^+^CD11b^+^ cells as shown in E. H) Representative dot plots of MDSCs (identified as CD11b^+^ cells gated as in B expressing F4/80 and intermediate levels of Gr-1) in the BALFs of VEH- and HDM-treated mice. I) Frequencies and J) absolute numbers of MDSCs as shown in H. K) Representative histograms showing PD-1 expression levels on CD11b^–^ cells gated as in A. L) Frequencies and M) absolute numbers of PD-1^+^CD11b^–^ cells as shown in K. Data are presented as mean ± SEM. Statistical significance was assessed by two-tailed Student’s t-test. n.s: non-significant, * *P* < 0.05, ** *P* < 0.01. **Supplemental Fig. 4.** Effect of HDM and other allergens on IL-1β secretion by BMDMs. A) Schematic overview of the BMDM culture system. Bone marrow (BM) cells were harvested from WT, *Nlrp3*, *Casp1*, or *Il1b* KO mice and were differentiated into BMDMs in presence of granulocyte-macrophage colony-stimulating factor (GM-CSF) for 7 days. BMDM (identified as CD11b^+^F4/80^+^ cells) purity was assessed by flow cytometry and was ~80% as shown in a representative dot plot. BMDMs were then stimulated for 24h with LPS, HDM, or other allergens, and ATP was added to each well for the last hour of culture. The supernatants were collected and the levels of IL-1β or TNF were analyzed by ELISA. B) WT BMDMs were pretreated for 1h at 37°C with an NLRP3 inhibitor (MCC950) or with a caspase-1 inhibitor (VX-765) at concentrations of 100 nM and 10 μM, respectively before being stimulated with HDM (200 μg/mL) and ATP (5 mM) as shown in A, and the levels of IL-1β or C) TNF in the supernatants were analyzed by ELISA. D) WT BMDMs were stimulated with the indicated concentrations of HDM *Dermatophagoides pteronyssinus* (DP) as in B and C, HDM *Dermatophagoides farinae* (DF), German cockroach (CR), ragweed pollen (RW), the fungi *Candida albicans* (CA) or *Alternaria alternata* (AA) and ATP (5 mM) as shown in A. The supernatants were collected and IL-1β production was analyzed by ELISA. IL-1β level in the supernatants of DP-treated cells was used as a reference and assigned to 100%. Data are representative of two (B and C) or three (D) independent experiments performed in triplicate and are presented as mean ± SEM. Statistical significance was assessed by one-way ANOVA with post hoc Bonferroni’s test. n.s: non-significant, * P < 0.05, ** *P* < 0.01, **** *P* < 0.0001. **Supplemental Fig. 5.** Effect of chronic exposure to HDM DF or ovalbumin on lung tumor development. A) *Kras*^*G12D*^ mice were treated i.n with VEH (*n* = 4), DF (*n* = 9), or ovalbumin (OVA, *n* = 6) as indicated in this schematic overview of the study design. B) Representative pictures of H&E-stained lung sections of mice in the three different groups. Scale bars, 2 mm. C) Tumor multiplicity calculated on H&E-stained sections as shown in B. Data are presented as mean ± SEM. Statistical significance was assessed by one-way ANOVA with post hoc Bonferroni’s test. n.s: non-significant, * *P* < 0.05, ** *P* < 0.01. **Supplemental Fig. 6.** Effect of IL-1β, CCL2, or NLRP3 neutralization in *Kras*^*G12D*^ mice. A) *Kras*^*G12D*^ mice were treated i.n with VEH or HDM and i.p with an anti-IL-1β, an anti-CCL2, or with the isotype control (ctrl) antibody (Ab), or with the NLRP3 inhibitor MCC950 as indicated in this schematic overview of the study design. B) Representative pictures of H&E-stained lung sections of mice treated with VEH + MCC950 (*n* = 7) or HDM + MCC950 (*n* = 11). Scale bars, 2 mm. C) Tumor multiplicity calculated on H&E-stained sections as shown in B. D) Tumors on H&E-stained sections as shown in B were classified into three grades (Grade 1, AAH and EH; Grade 2, AD; Grade 3, AC) and each grade was expressed as a percentage of the total. Data are representative of one experiment conducted in three independent cohorts pooled together (B–D) and are presented as mean ± SEM. Statistical significance was assessed by two-tailed Student’s t-test (C) or two-way ANOVA (D) with post hoc Bonferroni’s test. n.s: non-significant. **Supplemental Fig. 7.** HDM induces CCL2 production in the BALF of *Kras*^*G12D*^ mice and IL-1β neutralization inhibits this effect. A) ELISA analysis of CCL2 production in BALF recovered from *Kras*^*G12D*^ mice treated i.n with VEH, HDM, or HI-HDM as shown in Fig. 2A (n = 4 mice/group) or B) treated i.n with VEH or HDM and i.p with the anti-IL-1β or the isotype ctrl Ab as shown in Supplemental Fig. 6A (*n* = 3 mice/group). Data are representative of one experiment conducted twice independently and are presented as mean ± SEM. Statistical significance was assessed by one-way ANOVA with post hoc Bonferroni’s test. n.s: non-significant, * *P*< 0.05, ** *P* < 0.01, *** *P*< 0.001. **Supplemental Fig. 8.** Antibody validation for mIF and IHC staining. A) mIF staining of a mouse spleen section showing strong F4/80 and Ki-67 immunoreactivity. As expected, no PanCK was observed in the spleen. B) No background or non-specific binding was observed in the spleen negative control in the absence of the primary Abs. DAPI is shown for context. C) Positive control slide images using human tonsils showing strong PanCK and Ki-67 immunoreactivity. The F4/80 antibody does not work for human tissue. D) No background or non-specific binding was observed in the tonsil negative control in the absence of the primary Abs. DAPI is shown for context. E) Negative controls on a mouse lung section using secondary anti-Tag fluorescent Abs only. No staining was observed in absence of primary Abs. However, some autofluorescence was detected in the PanCK/CL550 (Cy3) channel from the heart tissue and red blood cells. Overlay (top) and single colors (bottom). DAPI nuclear staining (blue), F4/80 (magenta), Ki-67 (cyan) and PanCK (yellow). Scale bars, 0.25 mm (spleen and tonsil samples) and 2 mm (lung samples). F) IHC staining of a mouse lung lobe showing nuclear staining for TTF-1 in airway epithelial cells, including alveolar pneumocytes and club cells. No staining was observed in inflammatory cell infiltrates surrounding the bronchi and bronchioles. Scale bars, 1mm (upper panel) and 0.1 mm (inset). Data are representative of one (F) or two (A–G) independent experiments. **Supplemental Fig. 9.** Proposed model for the lung tumor-promoting effect of HDM. Chronic i.n instillation of HDM extracts triggers the activation of the NLRP3 inflammasome and caspase-1 in lung macrophages (MΦ, identified as CD11b^+^CD11c^–^F4/80^+^ cells), which leads to increased secretion of IL-1β in the lung microenvironment and persistent lung inflammation. In addition, HDM induces the accumulation of PD-1^+^-myeloid cells (identified as PD-1^+^CD11b^+^ cells) and myeloid-derived suppressor cells (MDSCs, identified as Gr-1^+^CD11b^+^ cells) in the lungs, which are known to inhibit anti-tumor immunity and to promote tumor cell proliferation. Neutralization of NLRP3, IL-1β, or CCL2, which could contribute to the recruitment of bone marrow (BM)-derived circulating monocytes and to the replenishment and pro-inflammatory phenotype of lung MΦ, as well as treatment with budesonide, which has anti-inflammatory and anti-proliferative properties, all inhibit the tumor-promoting effect of HDM. Finally, NLRP3 activation, IL-1β production, and the pro-tumorigenic effect of HDM were also significantly decreased but not completely abolished by heat treatment of the HDM extracts, suggesting that both heat-sensitive (e.g., proteolytic enzymes derived from the mites’ digestive system) and -insensitive (e.g., heat-insensitive proteases, LPS, and/or carbohydrates such as chitin) factors in HDM extract contributed to these effects. This schematic representation was created with BioRender.com and Servier Medical Art. **Supplemental Table 1.** Reagents used in this study. **Supplemental Table 2.** Oligonucleotides used for RT-qPCR analysis.

## Data Availability

The datasets used and/or analyzed during the current study are available from the corresponding author upon reasonable request.

## Abbreviations

AAH: Atypical adenomatous hyperplasia
Ab: Antibody
AC: Adenocarcinoma
AD: Adenoma
ATP: Adenosine triphosphate
BALF: Broncho-alveolar lavage fluid
BMDM: Bone marrow-derived macrophage
BW: Body weight
B6: C57BL/6 strain of mice
Casp-1: Caspase-1
CCSP: Clara cell secretory protein
CK: Cytokeratin
CLI: Chronic lung inflammation
DAPI: 4′,6-Diamidino-2-phenylindole blue-fluorescent DNA stain
DP: *Dermatophagoides pteronyssinus*
DF: *Dermatophagoides Farinae*
EGFR: Epidermal growth factor receptor
EH: Epithelial hyperplasia
ELISA: Enzyme-linked immunosorbent assay
EU: Endotoxin units
GM-CSF: Granulocytes macrophage colony-stimulating factor
H&E: Hematoxylin and eosin
HDM: House dust mites
HI: Heat-inactivated
ICS: Inhaled corticosteroid
IF: Immunofluorescence
IHC: Immunohistochemistry
IL: Interleukin
i.n: Intranasal/intranasally
i.p: Intraperitoneal/intraperitoneally
KO: Knockout
Kras: Kirsten rat sarcoma viral oncogene homolog
LC: Lung cancer
LPS: Lipopolysaccharide
MDSC: Myeloid-derived suppressor cell
MΦ: Macrophage
NF-κB: Nuclear factor-kappa B
NLPR3: Nod-like receptor family pyrin domain-containing protein 3
NSCLC: Non-small cell lung cancer
n.s: Non-significant
PAMPs: Pathogen-associated molecular patterns
PanCK: Pan-cytokeratin
PBS: Phosphate-buffered saline
PCNA: Proliferating cell nuclear antigen
PD-1: Programmed cell death protein 1
qPCR: Quantitative real-time PCR
RBC: Red blood cell
ROI: Region of interest
RT: Room temperature
SEM: Standard error of the mean
STAT3: Signal transducer and activator of transcription 3
TAM: Tumor-associated macrophage
TME: Tumor microenvironment
TNF: Tumor necrosis factor
VEGF: Vascular endothelial growth factor
VEH: Vehicle
