# Supplementary material for: Long-term exposure to house dust mites accelerates lung cancer development in mice
Source: J Exp Clin Cancer Res. 2023 Jan 21;42:26. doi: 10.1186/s13046-022-02587-9 (PMC9863279; doi:10.1186/s13046-022-02587-9)
Supplement: Supplementary file 1 — Additional file 1: Supplemental Fig. 1. Effect of chronic exposure to HDM in a urethane-induced LC model. A-F) WT C57BL/6 mice were treated i.n with HDM (n = 8), HI-HDM (n = 10), or with the vehicle (VEH, n = 9) and i.p with urethane as shown in Fig. 1A. A) Quantification of inflammatory cell infiltrates on H&E-stained lung sections as shown in Fig. 1D. B) The tumor multiplicity and C) the tumor area data presented in Fig. 1H–I were subdivided into male and female mice for the three experimental groups. D) Representative images of Wright-Giemsa staining of BALF cytospins. Magnification, 200x (overview panels), 400x (insets). E) BALF total and differential cell counts of BALF cytospins as shown in D. F) Correlation analysis of the number of monocytes/macrophages in the BALF with tumor multiplicity. The Pearson coefficient (R squared) and the corresponding P-value are shown. G) Representative pictures of H&E-stained lung sections of Rag1 KO mice treated i.n with VEH (n = 9) or HDM (n = 9), and i.p with urethane (0.6 mg/g of BW) as shown in Fig. 1A for WT mice. One lobe per mouse is shown. Arrowheads indicate tumors. Scale bars, 1 mm. H) Tumor multiplicity calculated on H&E-stained sections as shown in G. Data are presented as mean ± SEM. Statistical significance was assessed by one-way (A) or two-way (B, C, and E) ANOVA with post hoc Bonferroni’s test, linear regression using Pearson correlation (F), or two-tailed Student’s t-test (H). n.s: non-significant, * P < 0.05, ** P <0.01, *** P <0.001, **** P < 0.0001. Supplemental Fig. 2. Effect of chronic exposure to HDM in a KrasG12D-driven LC model. A) Five-week-old KrasG12D mice were treated i.n with VEH (n = 11) or HDM (n = 12) as indicated in this schematic overview of the study design. At 14 weeks of age and 72h after the last HDM challenge the mice were sacrificed and the BALFs and the lungs were harvested. B) Representative pictures of 4 lung lobes (dorsal view) of a VEH- and an HDM-treated mouse. Scale bars, 0.25 c [file 13046_2022_2587_MOESM1_ESM.pdf]

Supplemental Materials for

Long-Term Exposure to House Dust Mites Accelerates Lung Cancer Development in Mice

Dongjie Wang, PhD [1,2], Wen Li, PhD [1,3], Natalie Albasha, MS [1], Lindsey Griffin, BS [1], Han Chang, MS [1], Lauren Amaya, BS [1], Sneha Ganguly, MS [1], Liping Zeng, PhD [1,3], Bora Keum, MD [4], José M González-Navajas, PhD [5,6], Matt Levin, BS [7], Zohreh AkhavanAghdam, PhD [7], Helen Snyder, PhD [7], David Schwartz, BS [7], Ailin Tao, PhD [3], Laela M Boosherhri, MS[8], Hal M Hoffman, MD [8], Michael Rose, BS [9], Monica Valeria Estrada, MD [9], Nissi Varki, MD [10], Scott Herdman, BS [1], Maripat Corr, MD [1], Nicholas J.G Webster, PhD [11,12], Eyal Raz, MD [1*], Samuel Bertin, PhD [1*]

[1]Division of Rheumatology, Allergy & Immunology, Department of Medicine, University of California San Diego, La Jolla, California, USA

[2]Department of Pharmacology, Institute of Materia Medica, Chinese Academy of Medical Sciences & Peking Union Medical College, Beijing, China

[3]The Second Affiliated Hospital of Guangzhou Medical University, The State Key Laboratory of Respiratory Disease, Guangdong Provincial Key Laboratory of Allergy & Clinical Immunology, Center for Immunology, Inflammation and Immune-mediated Disease, Guangzhou, China

[4]Division of Gastroenterology & Hepatology, Department of Internal Medicine, Korea University College of Medicine, Seoul, Korea

[5]Networked Biomedical Research Center for Hepatic and Digestive Diseases (CIBERehd), Hospital General Universitario de Alicante, Alicante, Spain

[6]Alicante Institute of Health and Biomedical Research (ISABIAL), Alicante, Spain

[7]Cell IDx Inc., San Diego, California, USA

[8]Division of Pediatric Allergy, Immunology, and Rheumatology, Rady Children's Hospital of San Diego, University of California San Diego, La Jolla, California, USA

[9]Tissue Technology Shared Resource, Moores Cancer Center, University of California San Diego, La Jolla, California, USA

[10]Department of Pathology, University of California San Diego, 9500 Gilman Drive, La Jolla, California, USA

[11]Division of Endocrinology, Department of Medicine, University of California San Diego, 9500 Gilman Drive, La Jolla, California, USA

[12]Medical Research Service, Veteran Affairs San Diego Healthcare System, San Diego, California, USA

[*]Corresponding Authors:

Eyal Raz, Department of Medicine, University of California San Diego, 9500 Gilman Drive, La Jolla, CA 92093-0663, USA. Phone: 858-534-5444; Email: eraz@ucsd.edu

Samuel Bertin, Department of Medicine, University of California San Diego, 9500 Gilman Drive, La Jolla, CA 92093-0663, USA. Phone: 858-822-3758; Email: sbertin@ucsd.edu

This PDF file includes:

Supplemental Figures 1 to 9

Supplemental Tables 1 to 2

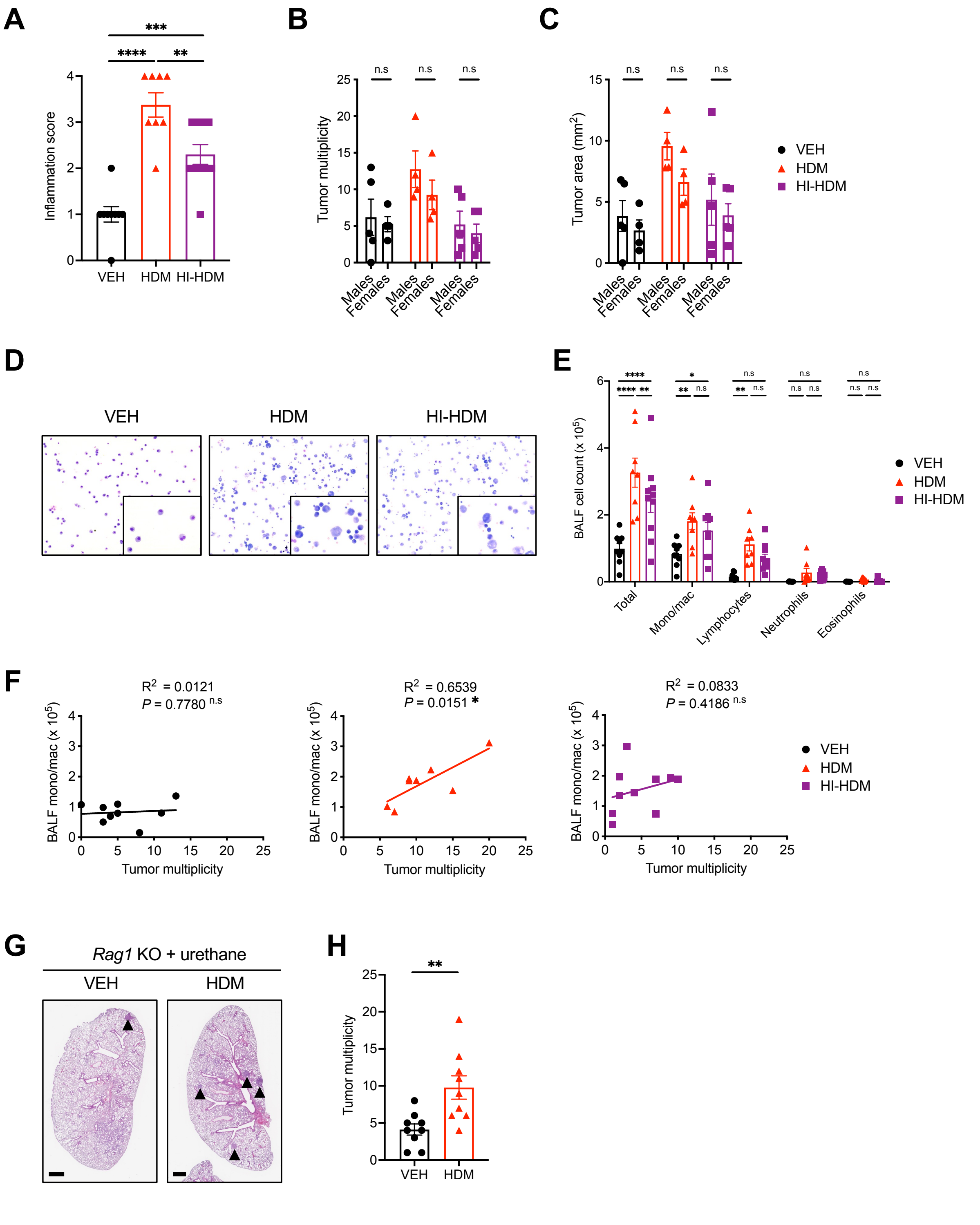

A
***
**** **
Inflammation score
VEH HDM HI-HDM
B
n.s n.s n.s
Tumor multiplicity
Males Females Males Females Males Females
C
n.s n.s n.s
Tumor area (mm²)
Males Females Males Females Males Females
VEH
HDM
HI-HDM
D
VEH HDM HI-HDM
E
BALF cell count (x 10⁵)
Total Mono/mac Lymphocytes Neutrophils Eosinophils
VEH
HDM
HI-HDM
F
R² = 0.0121
P = 0.7780 n.s
R² = 0.6539
P = 0.0151 *
R² = 0.0833
P = 0.4186 n.s
BALF mono/mac (x 10⁵)
Tumor multiplicity
VEH
HDM
HI-HDM
G
Rag1 KO + urethane
VEH HDM
H
**
Tumor multiplicity
VEH HDM

Supplemental Fig. 1. Effect of chronic exposure to HDM in a urethane-induced LC model. A-F) WT C57BL/6 mice were treated i.n with HDM (n = 8), HI-HDM (n = 10), or with the vehicle (VEH, n = 9) and i.p with urethane as shown in Fig. 1A. **A)** Quantification of inflammatory cell infiltrates on H&E-stained lung sections as shown in Fig. 1D. **B)** The tumor multiplicity and **C)** the tumor area data presented in Fig. 1H–I were subdivided into male and female mice for the three experimental groups. **D)** Representative images of Wright-Giemsa staining of BALF cytospins. Magnification, 200x (overview panels), 400x (insets). **E)** BALF total and differential cell counts of BALF cytospins as shown in D. **F)** Correlation analysis of the number of monocytes/macrophages in the BALF with tumor multiplicity. The Pearson coefficient (R squared) and the corresponding *P*-value are shown. **G)** Representative pictures of H&E-stained lung sections of *Rag1* KO mice treated i.n with VEH (n = 9) or HDM (n = 9), and i.p with urethane (0.6 mg/g of BW) as shown in Fig. 1A for WT mice. One lobe per mouse is shown. Arrowheads indicate tumors. Scale bars, 1 mm. **H)** Tumor multiplicity calculated on H&E-stained sections as shown in G. Data are presented as mean ± SEM. Statistical significance was assessed by one-way (A) or two-way (B, C, and E) ANOVA with post hoc Bonferroni's test, linear regression using Pearson correlation (F), or two-tailed Student's t-test (H). n.s: non-significant, * $P < 0.05$, ** $P < 0.01$, *** $P < 0.001$, **** $P < 0.0001$.

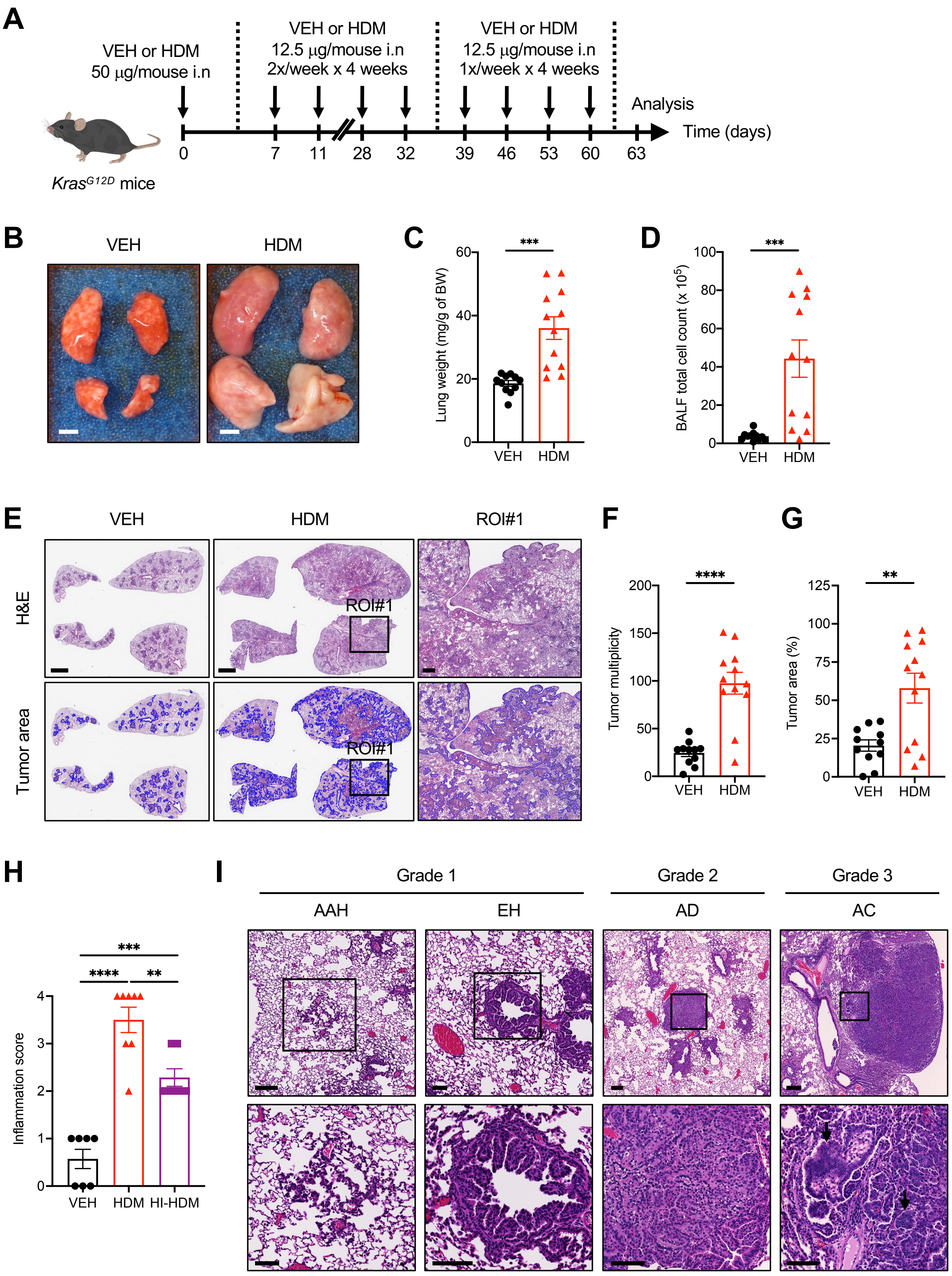
A
VEH or HDM
50 µg/mouse i.n
VEH or HDM
12.5 µg/mouse i.n
2x/week x 4 weeks
VEH or HDM
12.5 µg/mouse i.n
1x/week x 4 weeks
Analysis
Time (days)
0 7 11 28 32 39 46 53 60 63
$Kras^{G12D}$ mice
B
VEH
HDM
C
Lung weight (mg/g of BW)
VEH HDM
D
BALF total cell count (x 10^5)
VEH HDM
E
VEH
HDM
ROI#1
H&E
Tumor area
F
Tumor multiplicity
VEH HDM
G
Tumor area (%)
VEH HDM
H
Inflammation score
VEH HDM HI-HDM
I
Grade 1
Grade 2
Grade 3
AAH
EH
AD
AC

Supplemental Fig. 2. Effect of chronic exposure to HDM in a *Kras*^{G12D}-driven LC model. A) Five-week-old *Kras*^{G12D} mice were treated i.n with VEH (n = 11) or HDM (n = 12) as indicated in this schematic overview of the study design. At 14 weeks of age and 72h after the last HDM challenge the mice were sacrificed and the BALFs and the lungs were harvested. **B)** Representative pictures of 4 lung lobes (dorsal view) of a VEH- and an HDM-treated mouse. Scale bars, 0.25 cm. **C)** Lung weight normalized to mouse body weight (BW). **D)** BALF total cell counts. **E)** Representative pictures of H&E-stained lung sections. The lower panels are the same images as the above panels after tumor area quantification using QuPath software. The area within the blue borders was considered positive for lung tumors. The right panels show the boxed region (ROI#1) at higher magnification with areas of alveolar/bronchiolar hyperplasia and numerous mononuclear inflammatory infiltrates. Scale bars, 2 mm (whole lungs) and 0.5 mm (ROI#1). **F)** Tumor multiplicity calculated on H&E-stained sections as shown in E upper panels. **G)** Tumor area calculated on H&E-stained sections as shown in E lower panels. **H)** Quantification of inflammatory cell infiltrates on H&E-stained lung sections of 18-week-old *Kras*^{G12D} mice treated i.n with VEH, HDM, or HI-HDM as shown in Fig. 2E. **I)** Representative pictures of H&E-stained lung sections showing the different types of lung lesions observed in 18-week-old *Kras*^{G12D} mice treated i.n with VEH, HDM, or HI-HDM as shown in Fig. 2E. Top left panels: Grade 1 lesion of AAH with lepidic growth pattern observed in a *Kras*^{G12D} mouse treated with VEH. Scale bars, 0.1 mm (overview panel and inset). Middle left panels: Grade 1 lesion of EH of a respiratory bronchiole with hyperproliferative cells in the alveolar compartment observed in a *Kras*^{G12D} mouse treated with VEH. Scale bars, 0.1 mm (overview panel and inset). Middle right panels: Grade 2 AD of the papillary type with uniform nuclei observed in a *Kras*^{G12D} mouse treated with VEH. Scale bars, 0.2 mm (overview panel) and 0.1 mm (inset). Right panels: Grade 3 AC of the papillary

type with enlarged nuclei, prominent nucleoli, and some areas of tumor cell crowding (field of cells indicated by arrows) observed in a *Kras*^{G12D} mouse treated with HDM. Scale bars, 0.25 mm (overview panel) and 0.1 mm (inset). Data are representative of one experiment conducted in six independent cohorts of mice pooled together and are presented as mean ± SEM. Statistical significance was assessed by two-tailed Student's t-test (C, D, F, and G) or one-way ANOVA with post hoc Bonferroni's test (H). ** P <0.01, *** P <0.001, **** P <0.0001.

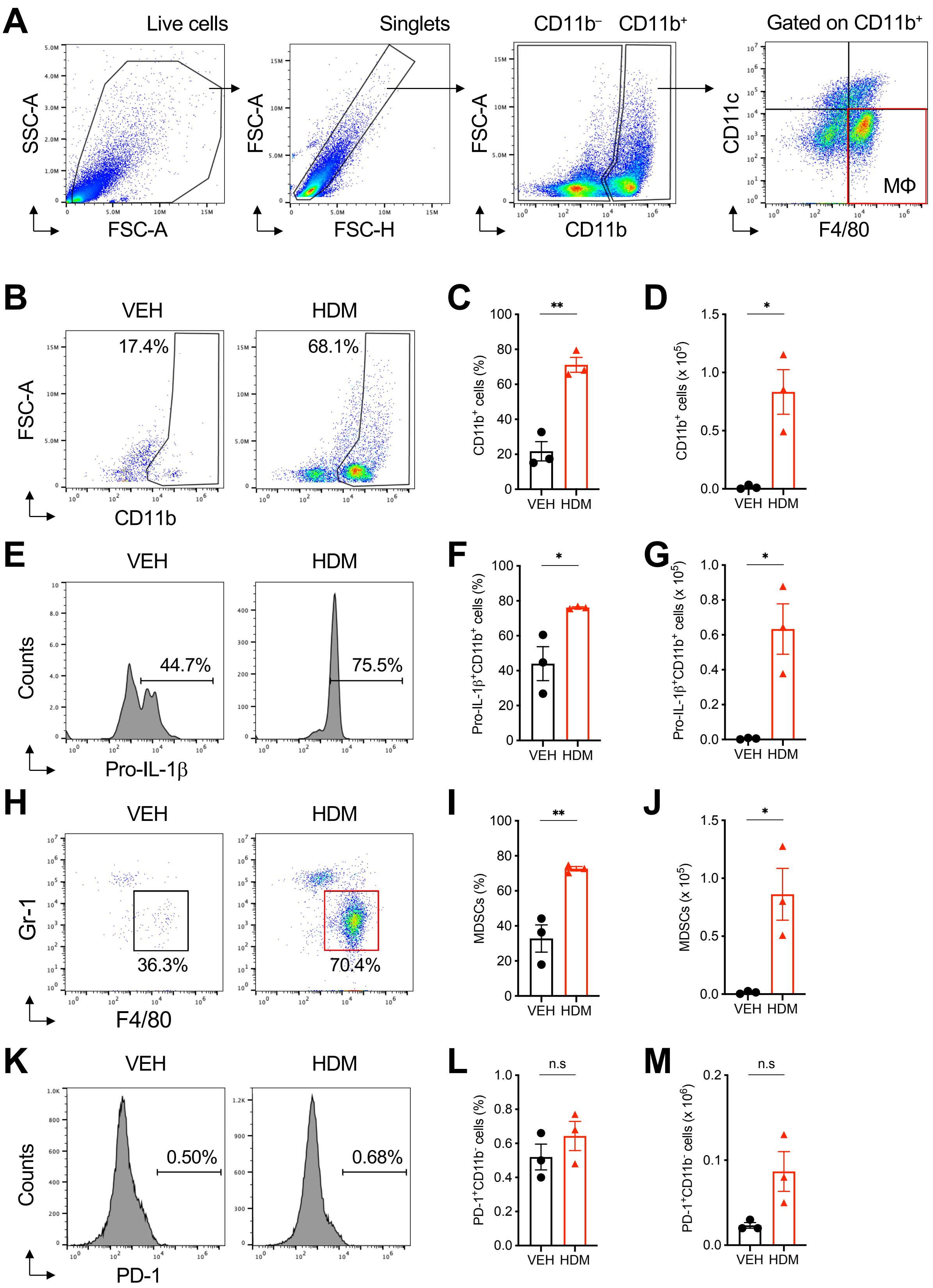
A
Live cells
Singlets
CD11b⁻ CD11b⁺
Gated on CD11b⁺
SSC-A
FSC-A
FSC-H
CD11b
CD11c
F4/80
MΦ
B
VEH
HDM
17.4%
68.1%
CD11b
C
CD11b⁺ cells (%)
D
CD11b⁺ cells (x 10⁵)
E
Counts
44.7%
75.5%
Pro-IL-1β
F
Pro-IL-1β⁺CD11b⁺ cells (%)
G
Pro-IL-1β⁺CD11b⁺ cells (x 10⁵)
H
Gr-1
36.3%
70.4%
F4/80
I
MDSCs (%)
J
MDSCs (x 10⁵)
K
0.50%
0.68%
PD-1
L
PD-1⁺CD11b⁻ cells (%)
M
PD-1⁺CD11b⁻ cells (x 10⁶)
n.s

Supplemental Fig. 3. Effect of chronic HDM exposure on the lung microenvironment. *Kras*^{G12D} mice were treated i.n with VEH or HDM (n = 3 mice/group) as shown in Fig. 2A and were sacrificed at day 61 (24h after the last i.n treatment). The lungs and the BALFs were recovered and single-cell suspensions were prepared for flow cytometry analyses. **A)** Representative flow cytometry plots of an HDM-treated mouse illustrating the gating strategy for the flow cytometry analyses. First, dead cells and debris were excluded based on forward scatter area (FSC-A) and side scatter area (SSC-A) values, then live single cells were gated based on forward scatter height (FSC-H) and FCS-A values. Macrophages (MΦ) were then identified as CD11b^{+}CD11c^{-}F4/80^{+} cells. **B)** Representative dot plots of cells expressing the pan-myeloid marker CD11b in the BALFs of VEH- and HDM-treated mice. **C)** Frequencies and **D)** absolute numbers of CD11b^{+} cells as shown in B. **E)** Representative histograms showing intracellular levels of pro-IL-1β in CD11b^{+} cells gated as in B in the BALFs of VEH- and HDM-treated mice. **F)** Frequencies and **G)** absolute numbers of pro-IL-1β^{+}CD11b^{+} cells as shown in E. **H)** Representative dot plots of MDSCs (identified as CD11b^{+} cells gated as in B expressing F4/80 and intermediate levels of Gr-1) in the BALFs of VEH- and HDM-treated mice. **I)** Frequencies and **J)** absolute numbers of MDSCs as shown in H. **K)** Representative histograms showing PD-1 expression levels on CD11b^{-} cells gated as in A. **L)** Frequencies and **M)** absolute numbers of PD-1^{+}CD11b^{-} cells as shown in K. Data are presented as mean ± SEM. Statistical significance was assessed by two-tailed Student's t-test. n.s: non-significant, * $P < 0.05$, ** $P < 0.01$.

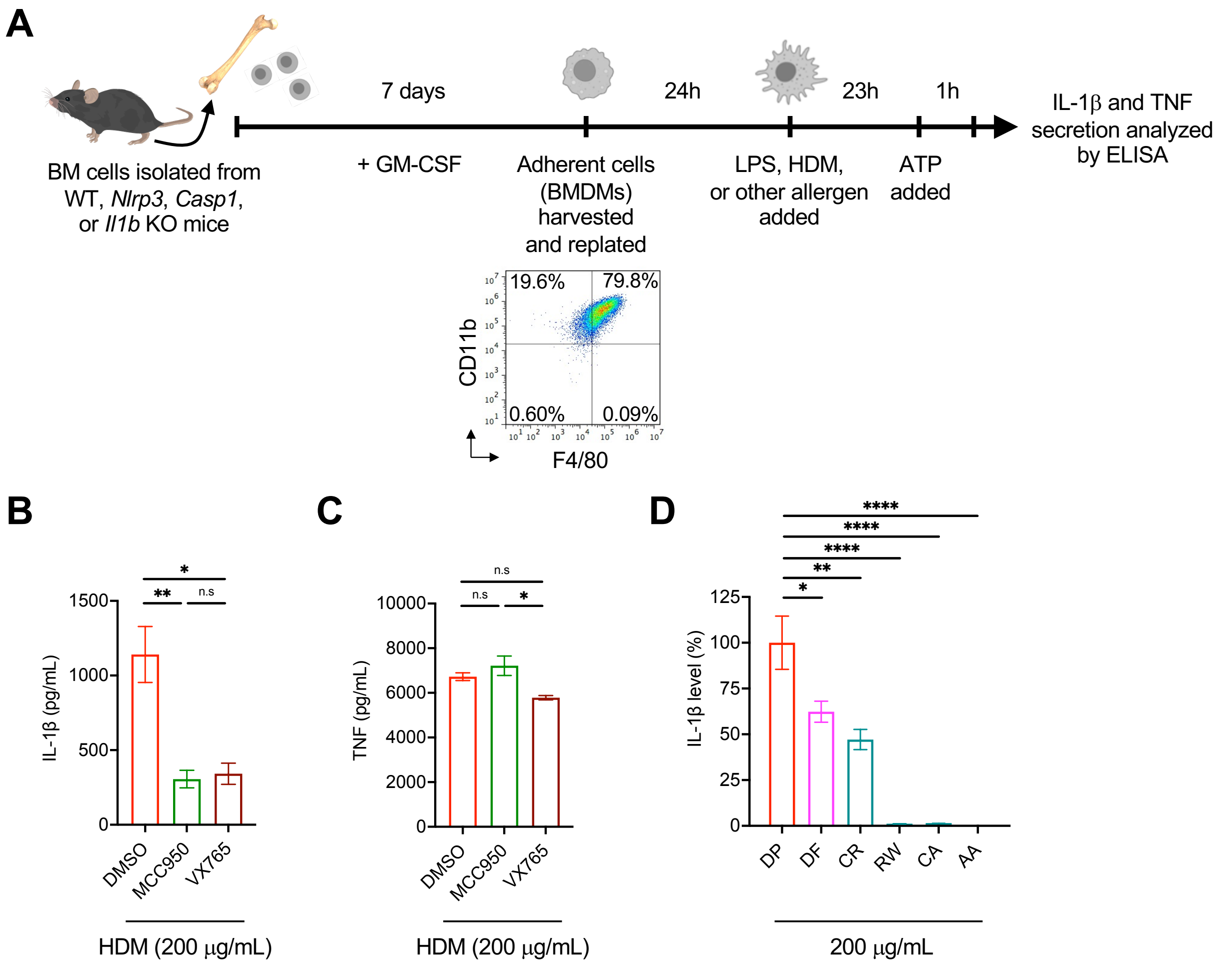

Supplemental Fig. 4. Effect of HDM and other allergens on IL-1β secretion by BMDMs. A) Schematic overview of the BMDM culture system. Bone marrow (BM) cells were harvested from WT, *Nlrp3*, *Casp1*, or *Il1b* KO mice and were differentiated into BMDMs in presence of granulocyte-macrophage colony-stimulating factor (GM-CSF) for 7 days. BMDM (identified as $CD11b^{+}F4/80^{+}$ cells) purity was assessed by flow cytometry and was ~80% as shown in a representative dot plot. BMDMs were then stimulated for 24h with LPS, HDM, or other allergens, and ATP was added to each well for the last hour of culture. The supernatants were collected and the levels of IL-1β or TNF were analyzed by ELISA. **B)** WT BMDMs were pretreated for 1h at 37°C with an NLRP3 inhibitor (MCC950) or with a caspase-1 inhibitor (VX-765) at concentrations of 100 nM and 10 µM, respectively before being stimulated with HDM (200 µg/mL) and ATP (5

mM) as shown in A, and the levels of IL-1β or **C)** TNF in the supernatants were analyzed by ELISA. **D)** WT BMDMs were stimulated with the indicated concentrations of HDM *Dermatophagoides pteronyssinus* (DP) as in B and C, HDM *Dermatophagoides farinae* (DF), German cockroach (CR), ragweed pollen (RW), the fungi *Candida albicans* (CA) or *Alternaria alternata* (AA) and ATP (5 mM) as shown in A. The supernatants were collected and IL-1β production was analyzed by ELISA. IL-1β level in the supernatants of DP-treated cells was used as a reference and assigned to 100%. Data are representative of two (B and C) or three (D) independent experiments performed in triplicate and are presented as mean ± SEM. Statistical significance was assessed by one-way ANOVA with post hoc Bonferroni's test. n.s: non-significant, * $P < 0.05$, ** $P < 0.01$, **** $P < 0.0001$.

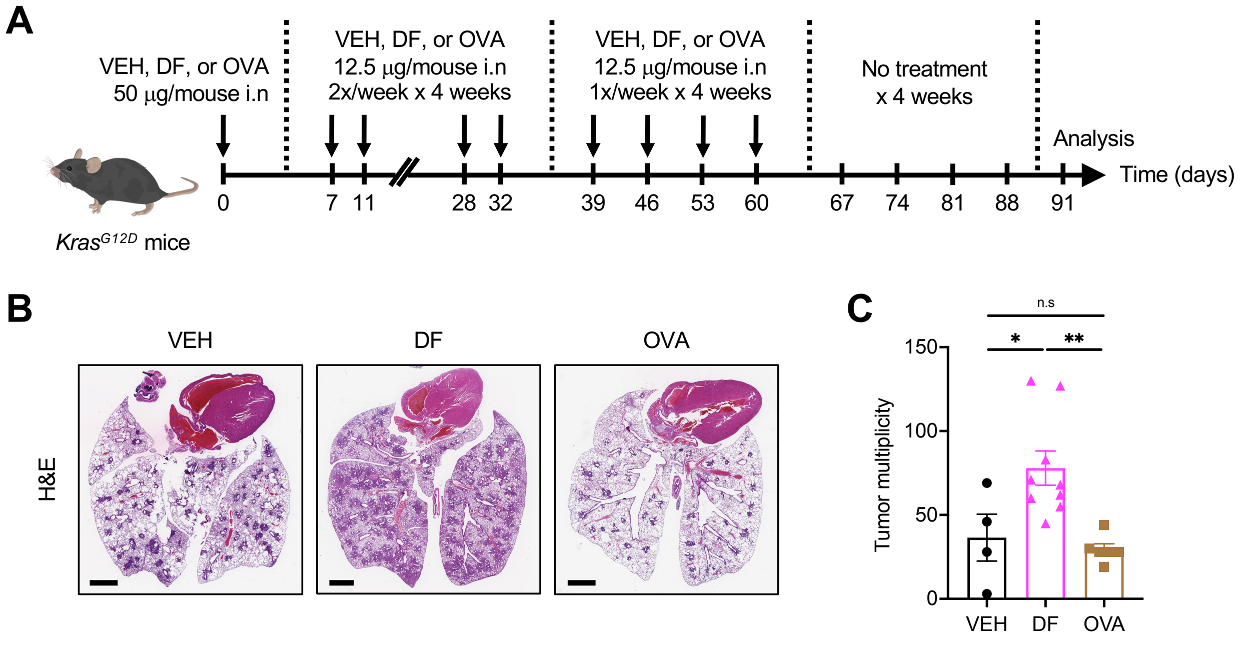

Supplemental Fig. 5. Effect of chronic exposure to HDM DF or ovalbumin on lung tumor development. A) *Kras*^{G12D} mice were treated i.n with VEH (n = 4), DF (n = 9), or ovalbumin (OVA, n = 6) as indicated in this schematic overview of the study design. **B)** Representative pictures of H&E-stained lung sections of mice in the three different groups. Scale bars, 2 mm. **C)** Tumor multiplicity calculated on H&E-stained sections as shown in B. Data are presented as mean ± SEM. Statistical significance was assessed by one-way ANOVA with post hoc Bonferroni's test. n.s: non-significant, * $P < 0.05$, ** $P < 0.01$.

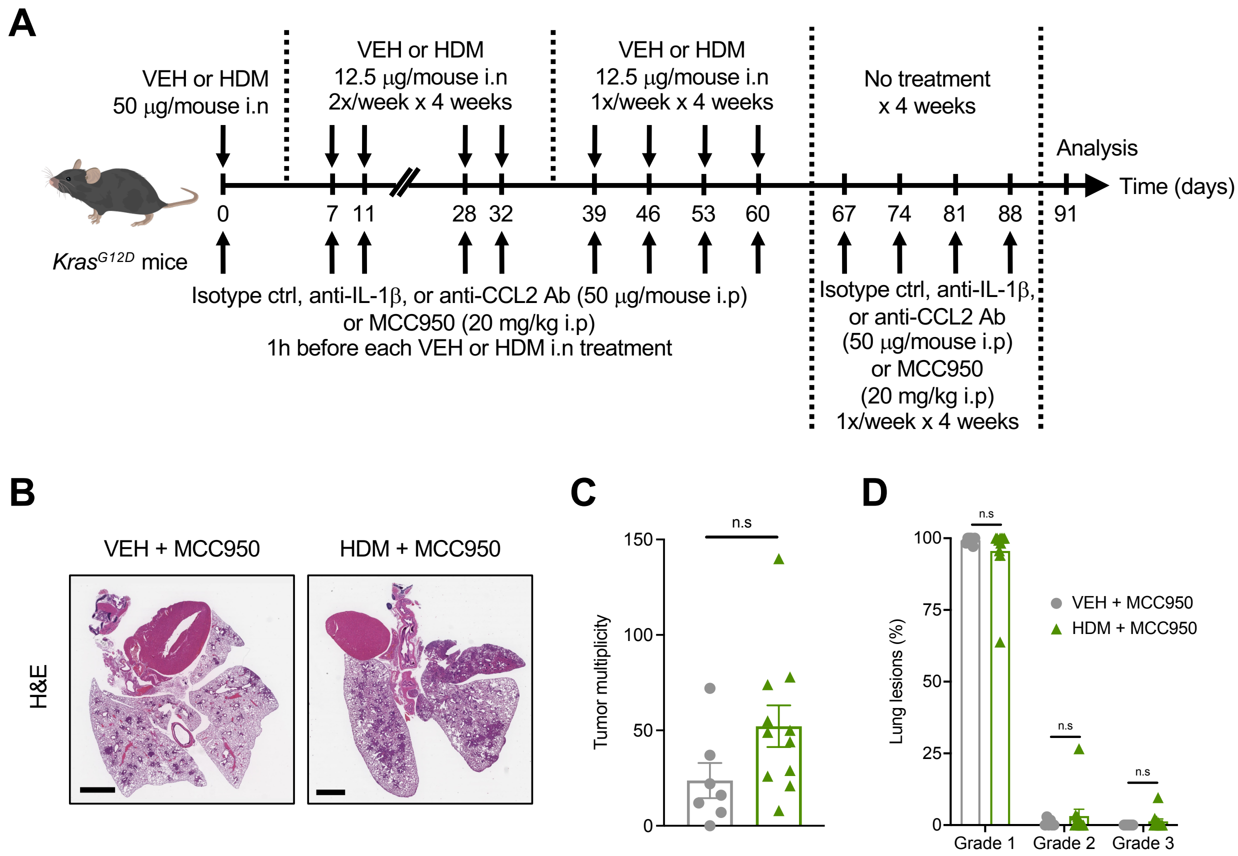

Supplemental Fig. 6. Effect of IL-1β, CCL2, or NLRP3 neutralization in *Kras*^{G12D} mice. A) *Kras*^{G12D} mice were treated i.n with VEH or HDM and i.p with an anti-IL-1β, an anti-CCL2, or with the isotype control (ctrl) antibody (Ab), or with the NLRP3 inhibitor MCC950 as indicated in this schematic overview of the study design. **B)** Representative pictures of H&E-stained lung sections of mice treated with VEH + MCC950 (n = 7) or HDM + MCC950 (n = 11). Scale bars, 2 mm. **C)** Tumor multiplicity calculated on H&E-stained sections as shown in B. **D)** Tumors on H&E-stained sections as shown in B were classified into three grades (Grade 1, AAH and EH; Grade 2, AD; Grade 3, AC) and each grade was expressed as a percentage of the total. Data are representative of one experiment conducted in three independent cohorts pooled together (B–D) and are presented as mean ± SEM. Statistical significance was assessed by two-tailed Student's t-test (C) or two-way ANOVA (D) with post hoc Bonferroni's test. n.s: non-significant.

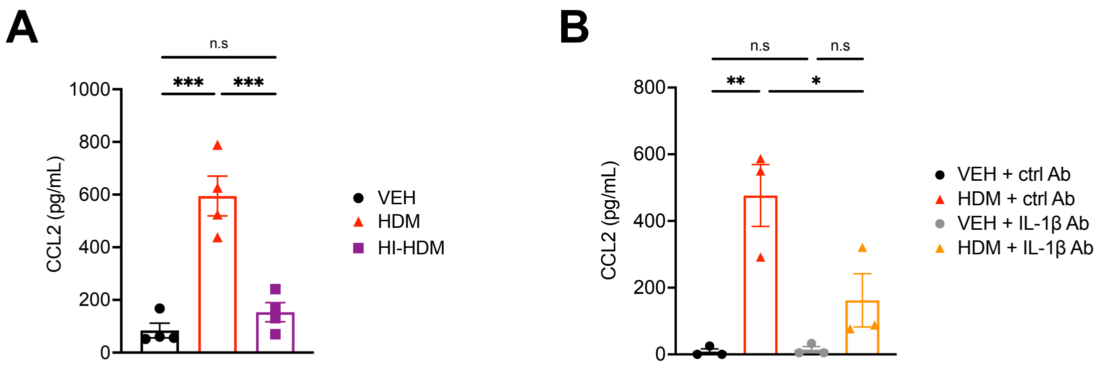

Supplemental Fig. 7. HDM induces CCL2 production in the BALF of *Kras*^{G12D} mice and IL-1β neutralization inhibits this effect. A) ELISA analysis of CCL2 production in BALF recovered from *Kras*^{G12D} mice treated i.n with VEH, HDM, or HI-HDM as shown in Fig. 2A (n = 4 mice/group) or **B)** treated i.n with VEH or HDM and i.p with the anti-IL-1β or the isotype ctrl Ab as shown in Supplemental Fig. 6A (n = 3 mice/group). Data are representative of one experiment conducted twice independently and are presented as mean ± SEM. Statistical significance was assessed by one-way ANOVA with post hoc Bonferroni's test. n.s: non-significant, * $P < 0.05$, ** $P < 0.01$, *** $P < 0.001$.

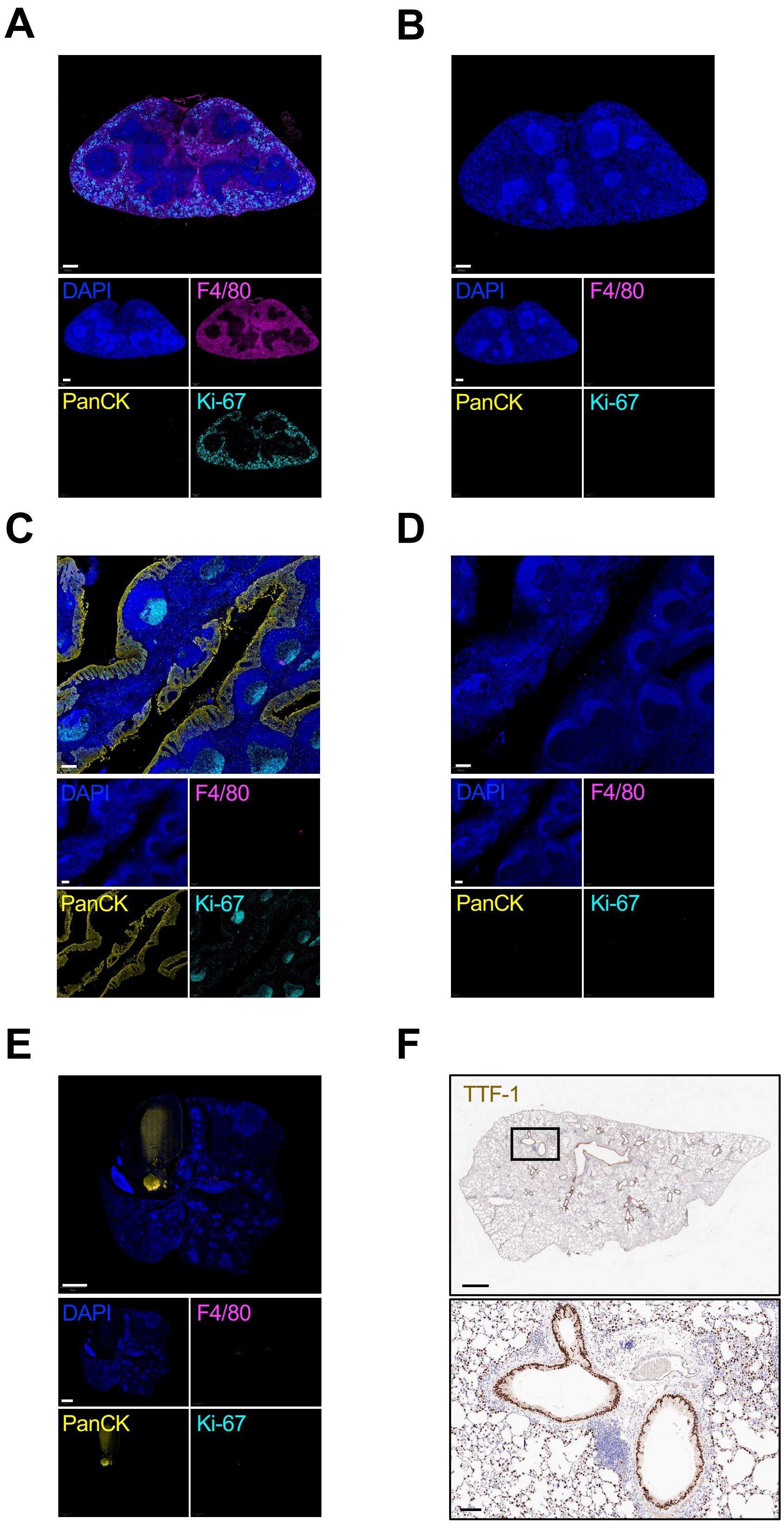
A
B
C
D
E
F
DAPI
F4/80
PanCK
Ki-67
TTF-1

Supplemental Fig. 8. Antibody validation for mIF and IHC staining. A) mIF staining of a mouse spleen section showing strong F4/80 and Ki-67 immunoreactivity. As expected, no PanCK was observed in the spleen. **B)** No background or non-specific binding was observed in the spleen negative control in the absence of the primary Abs. DAPI is shown for context. **C)** Positive control slide images using human tonsils showing strong PanCK and Ki-67 immunoreactivity. The F4/80 antibody does not work for human tissue. **D)** No background or non-specific binding was observed in the tonsil negative control in the absence of the primary Abs. DAPI is shown for context. **E)** Negative controls on a mouse lung section using secondary anti-Tag fluorescent Abs only. No staining was observed in absence of primary Abs. However, some autofluorescence was detected in the PanCK/CL550 (Cy3) channel from the heart tissue and red blood cells. Overlay (top) and single colors (bottom). DAPI nuclear staining (blue), F4/80 (magenta), Ki-67 (cyan) and PanCK (yellow). Scale bars, 0.25 mm (spleen and tonsil samples) and 2 mm (lung samples). **F)** IHC staining of a mouse lung lobe showing nuclear staining for TTF-1 in airway epithelial cells, including alveolar pneumocytes and club cells. No staining was observed in inflammatory cell infiltrates surrounding the bronchi and bronchioles. Scale bars, 1mm (upper panel) and 0.1 mm (inset). Data are representative of one (F) or two (A–G) independent experiments.

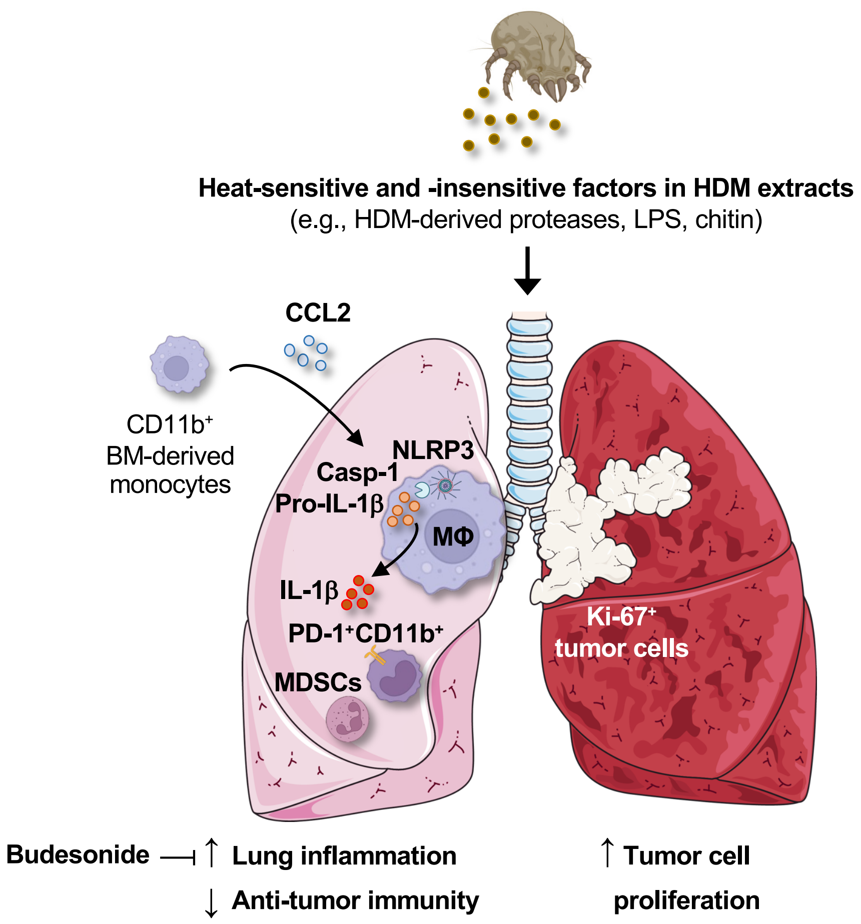

Supplemental Fig. 9. Proposed model for the lung tumor-promoting effect of HDM. Chronic i.n instillation of HDM extracts triggers the activation of the NLRP3 inflammasome and caspase-1 in lung macrophages (MΦ, identified as $CD11b^{+}CD11c^{-}F4/80^{+}$ cells), which leads to increased secretion of IL-1β in the lung microenvironment and persistent lung inflammation. In addition, HDM induces the accumulation of $PD\text{-}1^{+}$-myeloid cells (identified as $PD\text{-}1^{+}CD11b^{+}$ cells) and myeloid-derived suppressor cells (MDSCs, identified as $Gr\text{-}1^{+}CD11b^{+}$ cells) in the lungs, which are known to inhibit anti-tumor immunity and to promote tumor cell proliferation. Neutralization of NLRP3, IL-1β, or CCL2, which could contribute to the recruitment of bone marrow (BM)-derived circulating monocytes and to the replenishment and pro-inflammatory phenotype of lung MΦ, as well as treatment with budesonide, which has anti-inflammatory and anti-proliferative

properties, all inhibit the tumor-promoting effect of HDM. Finally, NLRP3 activation, IL-1β production, and the pro-tumorigenic effect of HDM were also significantly decreased but not completely abolished by heat treatment of the HDM extracts, suggesting that both heat-sensitive (e.g., proteolytic enzymes derived from the mites' digestive system) and -insensitive (e.g., heat-insensitive proteases, LPS, and/or carbohydrates such as chitin) factors in HDM extract contributed to these effects. This schematic representation was created with BioRender.com and Servier Medical Art.

Supplemental Table 1. Reagents used in this study.

Reagent	Supplier	Identifier
Antibodies		
Rat anti-mouse/human CD11b-PE/Cyanine7	BioLegend	Cat# 101216, RRID:AB_312799
Rat anti-mouse F4/80-PE	BioLegend	Cat# 123110, RRID:AB_893486
Rat anti-mouse Ly-6G/Ly-6C (Gr-1)-APC	BioLegend	Cat# 108412, RRID:AB_313377
Armenian hamster anti-mouse CD11c-APC	BioLegend	Cat# 117310, RRID:AB_313779
Rat anti-mouse CD16/CD32 (Fc Block)	BioLegend	Cat# 101320, RRID:AB_1574975
Armenian hamster anti-mouse CD279 (PD-1)-PE	Thermo Fisher Scientific	Cat# 12-9985-81, RRID:AB_466294
Rat anti-mouse IL-1β (Pro-form)-FITC	Thermo Fisher Scientific	Cat# 11-7114-80, RRID:AB_10718839
Rabbit anti-mouse IL-1β	Cell Signaling Technology	Cat# 12426, RRID:AB_2797907
Armenian hamster anti-mouse/rat/hamster IL-1β	Santa Cruz Biotechnology	Cat# sc-12742, RRID:AB_627791
Mouse anti-mouse/rat caspase-1 p20	Santa Cruz Biotechnology	Cat# sc-398715, RRID:AB_2819181
Rabbit anti-mouse/human NLRP3	Cell Signaling Technology	Cat# 15101, RRID:AB_2722591
Mouse anti-mouse β-Actin	Sigma-Aldrich	Cat# A5316, RRID:AB_476743
Goat anti-rabbit IgG-HRP	Cell Signaling Technology	Cat# 7074, RRID:AB_2099233
Goat anti-mouse IgG-HRP	BioLegend	Cat# 405306, RRID:AB_315009
Mouse anti-Armenian hamster IgG-HRP	Santa Cruz Biotechnology	Cat# sc-2789, RRID:AB_628484
Armenian hamster IgG control	BioXCell	Cat# BE0091, RRID:AB_1107773
Armenian hamster anti-mouse/rat IL-1β	BioXCell	Cat# BE0246, RRID:AB_2687727
Armenian hamster anti-mouse/rat/human CCL2 (MCP-1)	BioXCell	Cat# BE0185, RRID:AB_10950302
Rabbit anti-mouse/rat/human TTF-1	Abcam	Cat# ab76013, RRID:AB_1310784
Goat anti-rabbit IgG-HRP	Cell IDx	Cat# 2RH-100
Mouse anti-mouse/human panCK-UT016	Cell IDx	Cat# Anti Mse panCK-UT016, Clone# AE1/AE3, Lot# C41-01-P1
Rabbit anti-mouse F4/80-UT021	Cell IDx	Cat# Anti Mse F4/80-UT021, Clone# SP115, Lot# C35-210-P1
Mouse anti-mouse/human Ki-67-UT019	Cell IDx	Cat# Anti Mse Ki-67-UT019, Clone# B56, Lot# C35-214-P2

Rabbit anti-UT016 CellLight (CL)-550	Cell IDx	Cat# Anti UT016-CL550, Lot# C42-45-P1
Rabbit anti-UT021 CellLight (CL)-650	Cell IDx	Cat# Anti UT021-CL650, Lot# C35-136-P3
Rabbit anti-UT019 CellLight (CL)-750	Cell IDx	Cat# Anti UT019-CL750, Lot# C42-35-P2
Chemical compounds		
Urethane	Sigma-Aldrich	Cat# U2500
HDM *Dermatophagoides pteronyssinus* (DP) extract	Greer laboratories	Cat# XPB82D3A2.5 or XPB82D3A25
HDM *Dermatophagoides farinae* (DF) extract	Greer laboratories	Cat# XPB81D3A2.5
German cockroach (CR) extract	Greer laboratories	Cat# XPB46D3A4
Ragweed pollen (RW) extract	Greer laboratories	Cat# XP56D3A2.5
Candida albicans (CA) extract	Greer laboratories	Cat# XPM15D3A5
Alternaria alternata (AA) extract	Greer laboratories	Cat# XPM1D3A2.5
Ovalbumin (OVA)	Worthington Biochemical	Cat# LS003054
Budesonide	Tocris	Cat# 2671
Lipopolysaccharide (LPS)	Sigma-Aldrich	Cat# L2630
Adenosine 5′-triphosphate (ATP) disodium salt	Sigma-Aldrich	Cat# A7699
IC Fixation Buffer	eBioscience	Cat# 00-8222-49
Permeabilization Buffer	eBioscience	Cat# 00-8333-56
Collagenase type IA	Sigma-Aldrich	Cat# C9891
DNAse I	Sigma-Aldrich	Cat# D5025
Penicillin-Streptomycin	Sigma-Aldrich	Cat# P4333
Cytoseal 60	Thermo Fisher Scientific	Cat# 23-244257
VX-765	Selleckchem	Cat# S2228
MCC950	Selleckchem	Cat# S7809
3,3'-Diaminobenzidine (DAB) Chromogen	VWR	Cat# 95041-478
Mayer’s Hematoxylin	Sigma-Aldrich	Cat# 51275
Hematoxylin	Thermo Fisher Scientific	Cat# 7221
Eosin	Thermo Fisher Scientific	Cat# 7111
Wright-Giemsa Stain	Thermo Fisher Scientific	Cat# 23-264983
Antigen Unmasking Solution	Vector Labs	Cat# H-3301
InVivoPure pH 7.0 Dilution Buffer	BioXCell	Cat# IP0070
Recombinant mouse GM-CSF	BioLegend	Cat# 576302
Commercial assays		
Mouse IL-1β ELISA kit	R&D Systems	Cat# DY401-05
Mouse TNF ELISA kit	Thermo Fisher Scientific	Cat# 88-7324-88
Mouse CCL2 ELISA kit	Thermo Fisher Scientific	Cat# 88-7391-88
PureLink RNA Mini Kit	Thermo Fisher Scientific	Cat# 12183018A
qScript cDNA SuperMix	Quanta Biosciences	Cat# 95048
PowerUp SYBR Green Master Mix	Thermo Fisher Scientific	Cat# A25742
Cell lines		
RAW 264.7 cells	ATCC	Cat# TIB-71, RRID:CVCL_0493

Mice		
WT C57BL/6: C57BL/6J	The Jackson Laboratory	RRID:IMSR_JAX:000664
Nlrp3 KO: B6.129S6-*Nlrp3*^{tm1Bhk}/J	The Jackson Laboratory	RRID:IMSR_JAX:021302
Casp1 KO: B6N.129S2-^{Casp1tm1Flv}/J	The Jackson Laboratory	RRID:IMSR_JAX:016621
IL1b KO: C;129S2-*Il1b*^{tm1Dch}/MerJ	The Jackson Laboratory	RRID:IMSR_JAX:034447
Rag1 KO: B6.129S7-*Rag1*^{tm1Mom}/J	The Jackson Laboratory	RRID:IMSR_JAX:002216
LSL-Kras^{G12D}: B6.129S4-*Kras*^{tm4Tyj}/J	The Jackson Laboratory	RRID:IMSR_JAX:008179
CCSP^{Cre}: B6.129-*Scgb1a1*$^{tm1(icre)Fjd}$/J	The Jackson Laboratory	RRID:IMSR_JAX:036525
Oligonucleotides		
qPCR primers, see Supplemental Table 2	Integrated DNA Technologies	N/A
Software		
ObjectiveView	Objective Pathology	https://www.objectivepathology.com/objectiveview
QuPath v0.2.0	Open Source	RRID:SCR_018257; https://qupath.github.io/
Image-Pro Premier 10.0	Media Cybernetics	RRID:SCR_016497; http://www.mediacy.com/imagepro
Prism v9.0	GraphPad Software	RRID:SCR_002798; https://www.graphpad.com/
FlowJo v10.7.1	Tree Star	RRID:SCR_008520; https://www.flowjo.com/
ImageJ 1.53a	National Institute of Health	RRID:SCR_003070; https://imagej.nih.gov/ij/
ImageLab	Bio-Rad	https://www.bio-rad.com/
BioRender	BioRender	RRID:SCR_018361; https://biorender.com/
Other		
Cytospin 2	Shandon	N/A
Accuri C6 flow cytometer	BD biosciences	N/A
QuantStudio 3 Real-Time PCR System	Thermo Fisher Scientific	Cat# A28137
Leica Aperio AT2 slide scanner	Leica Biosystems	RRID:SCR_021256
Leica Aperio VERSA slide scanner	Leica Biosystems	RRID:SCR_021016
Hamamatsu Nanozoomer slide scanner	Hamamatsu Photonics	N/A
Gemini AS slide stainer	Thermo Fisher Scientific	N/A

Supplemental Table 2. Oligonucleotides used for RT-qPCR analysis.

	**qPCR primer forward**	**qPCR primer reverse**
Nlrp1	CCACTGAGCTACTATGCAGTACA	ACAACATCTTCACACCACCATC
Nlrp3	ATCAACAGGCGAGACCTCTG	GTCCTCCTGGCATACCATAGA
Nlrc4	CTTGGCCAGGAGAGCCTTG	GGGCTCGTCTGTTGTTCCTT
Nlrp6	CCCTCACACCCAGAATGAGAC	GCATCCACGCTGGAGCA
Nlrp12	CGACCCACCAGAACCTTCAG	CAGGCAGCATGTTCCTTTCC
Il1b	GCAACTGTTCCTGAACTCAACT	ATCTTTTGGGGTCCGTCAACT
Myc	TTGGAAACCCCGCAGACAG	GCTGTACGGAGTCGTAGTCG
Ctnnb1	GTTGACACCTCCCAAGTCCT	CTGCCCGTCAATATCAGCTA
Ccnd1	TGACTGCCGAGAAGTTGTGC	CTCATCCGCCTCTGGCATT
Mki67	AAGTCTCTTGGCACTCACAG	TGCCTGATCTGCGTCTTTG
Pcna	TAAAGAAGAGGAGGCGGTAA	TAAGTGTCCCATGTCAGCAA
Krt19	GACCTAGCCAAGATCCTGAGT	TCAGCTCCTCAATCCGAGCA
Gapdh	TCAACAGCAACTCCCACTCTT	ACCCTGTTGGTGTAGCCGTAT
